# Inverse problems in blood flow modeling: A review

**DOI:** 10.1002/cnm.3613

**Published:** 2022-05-24

**Authors:** David Nolte, Cristóbal Bertoglio

**Affiliations:** ^1^ Bernoulli Institute University of Groningen Groningen The Netherlands; ^2^ Center for Mathematical Modeling Universidad de Chile Santiago Chile; ^3^ Department of Fluid Dynamics Technische Universität Berlin Berlin Germany

**Keywords:** blood flows, inverse problems, mathematical modeling, medical imaging

## Abstract

Mathematical and computational modeling of the cardiovascular system is increasingly providing non‐invasive alternatives to traditional invasive clinical procedures. Moreover, it has the potential for generating additional diagnostic markers. In blood flow computations, the personalization of spatially distributed (i.e., 3D) models is a key step which relies on the formulation and numerical solution of inverse problems using clinical data, typically medical images for measuring both anatomy and function of the vasculature. In the last years, the development and application of inverse methods has rapidly expanded most likely due to the increased availability of data in clinical centers and the growing interest of modelers and clinicians in collaborating. Therefore, this work aims to provide a wide and comparative overview of literature within the last decade. We review the current state of the art of inverse problems in blood flows, focusing on studies considering fully dimensional fluid and fluid–solid models. The relevant physical models and hemodynamic measurement techniques are introduced, followed by a survey of mathematical data assimilation approaches used to solve different kinds of inverse problems, namely state and parameter estimation. An exhaustive discussion of the literature of the last decade is presented, structured by types of problems, models and available data.

## Graphical abstract

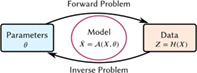

## 1 INTRODUCTION

Cardiovascular disease (CVD) is the major cause of death globally.^1^ Alone in Europe, CVD causes 3.9 million deaths pear year, accounting for 45% of deaths from all causes. The estimated overall cost of CVD for the economy of the European Union is €210 billion.^2^

CVDs generally alter the blood circulation by redirection or obstruction of the blood flow due to malformations of the heart, vessels or heart valves, by modification of the tissue properties (e.g., stiffness, lesions) or due to building up of arterial plaques. Accordingly, the diagnosis of CVD assesses hemodynamic properties of the diseased vessels. For instance, valvular stenosis, narrowing of vessels due to congenital abnormalities or atherosclerosis can cause oscillatory flow disturbances and turbulence leading to an *increase in hemodynamic pressure gradient* and thus an increased cardiac load. Therefore, the pressure gradient is extensively used as a diagnostic indicator of the severity of blood flow obstructions.^3^, ^4^, ^5^, ^6^, ^7^

Another example is the assessment of the *arterial wall stiffness* (AWS), which is an extremely important indicator for the early diagnosis of hypertension,^8^ affecting around 20% of the population and with 40% the risk of developing lethal CVD.^9^ For instance, aortic aneurysms affect 4.8% of the overall population and have a 70% mortality rate after rupture.^10^ Recent evidence suggests that aneurysms rupture is most likely to occur in stiffer arteries.^11^ Therefore, evaluating the progression of AWS in aortic aneurysms would allow to develop early indicators of disease severity evolution before the onset of irreversible pathological events.

Unfortunately, the hemodynamic quantities assessable by means of clinical measurement (such as medical imaging) are limited and the techniques not always non‐invasive. However, mathematical approaches for formulating and solving inverse problems in hemodynamics have the capacity to extract and estimate unobserved quantities of interest from (non‐invasive) hemodynamic measurements and can, for instance, reconstruct incomplete datasets and enrich or denoise measurements. This work aims at providing a wide overview of the recent advances of mathematical formulations and computational methods concerned with inverse problems in hemodynamics. The formulation and solution of these problems will depend heavily on the quantity of interest and the type of data available.

To the best of our knowledge, the first review on image‐based blood flow modeling was published in 2010 by Taylor and Steinman.^12^ This review, however, did not consider model personalization in terms of solutions of inverse problems but rather of direct imposition of measurements (e.g., geometry, flow rate and pressure forms) in the model. The reason is that, at that time, inverse hemodynamics was just starting as a research field. The first work reviewing and summarizing results concerning variational data assimilation (for both boundary conditions in stationary flows and stiffness in fluid–solid interaction was reported in 2012 by D'Elia et al.^13^ which was later extended by Veneziani and Vergara.^14^ A year later, Marsden^15^ reviewed optimization problems and approaches in cardiovascular flows, with a part devoted to the estimation of properties of numerical flow models from data. The most recent effort in reviewing inverse hemodynamics methods and applications was carried out in 2017 and 2019 by Quarteroni et al.^16^, ^17^ Here, detailed descriptions of the methodologies for variational and sequential parameter estimation approaches are given, while the review of published research proceeds until 2014. Since then, an important number of interesting publications have appeared leading to major advances in the field in terms of methodologies and applications. Therefore, the aim of this work is to present an updated overview of the current state‐of‐the‐art approaches from a *modeler*'s perspective. In particular, we analyze and discuss the outcomes of each of the works in detail with the aim of revealing the research gaps requiring future scientific developments.

The remainder article is organized as follows:

- Section 2 presents the modeling assumptions and equations used for blood flow modeling.
- Section 3 summarizes the measurement techniques typically used to study blood flows.
- Section 4 reviews the methods for directly computing pressure gradients from full field velocity measurements.
- Section 5 gives a background on the mathematical methods used to solve inverse problems in hemodynamics.
- Section 6 reviews the research on the estimation of distributed boundary conditions in rigid wall models.
- Section 7 reviews the research on the estimation of reduced‐order model parameters serving as boundary conditions in rigid wall models.
- Section 8 reviews the research on the estimation of material properties in fluid–structure interaction models.
- Section 9 reviews the research on compensating errors in the computational geometry.
- Section 10 reviews the research on state estimation concerned with errors in the initial condition in both rigid wall and fluid–structure interaction models.
- Section 11 briefly lists works on further related topics which are not exhaustively covered in this article.

## 2 BLOOD FLOW MODELS

### 2.1 Full‐dimensional description

Blood is a suspension of formed elements (i.e., red and white blood cells, platelets) in plasma.^18^ In hemodynamics—the macroscopic description of the dynamics of blood flow through the vessels—blood is considered a continuous single‐phase fluid (under the continuum hypothesis^19^). Blood acts as a non‐Newtonian fluid with viscoelastic behavior, originating from the deformability of the red blood cells. Its apparent viscosity depends on the viscosity of the plasma (a Newtonian fluid), the hematocrit (volume fraction of blood cells in the blood), red blood cell mechanical properties and red blood cell aggregation.^18^

Under the continuum hypothesis, blood flow is assumed to satisfy linear momentum, angular momentum and mass conservation. These physical laws are mathematically represented by the incompressible Navier–Stokes equations:

(1) $$\begin{array}{r} \rho \frac{\partial u}{\partial t}+\rho \left(u\cdot \nabla \right)u+\nabla p-\nabla \cdot \tau =0\\ \nabla \cdot u=0 \end{array}$$

with the velocity vector $u:\Omega \times T\mapsto {\mathbb{R}}^{3}$, the pressure p:Ω×T↦ℝ, in a spatial domain Ω (a subset of the blood vessel of interest) and a time interval *T*, and neglecting the gravitational force. The constant *ρ* > 0 denotes then mass density of the fluid, and *τ* denotes the viscous stress tensor and is determined by a constitutive equation modeling the shear behavior of blood. Classical models accurately describing the non‐Newtonian rheology of blood are the Casson and the Carreau‐Yasuda models.^20^, ^21^ At high shear rates and moderate‐to‐high Reynolds numbers blood behaves approximately as a Newtonian fluid.^22^, ^23^ It is often assumed that such conditions exist in the flow through large vessels.^24^ Under the assumption of a Newtonian fluid, the viscous stress tensor becomes

(2) $$\tau =\mu \left(\nabla u+{\left(\nabla u\right)}^{T}\right)$$

with the constant dynamic viscosity, *μ*. When this assumption is acceptable is a question of on‐going debate and it has been shown to be inaccurate in some situations.^21^ This assumptions is the most used in inverse problems described later.

In order to solve Equation (1), it needs to be complemented with appropriate boundary and initial conditions ***u*** (*t*_0_) = ***u***_0_. So‐called Dirichlet boundary conditions impose a given velocity profile ***u***_*D*_ on the whole or a part of the boundary Γ_*D*_ ⊂ *∂*Ω, and Neumann boundary conditions specify the normal stress vector ***g***_*N*_ = ***τn*** − *p**n*** (***n*** is the outward normal vector) on the remainder of the boundary Γ_*N*_ = *∂*Ω ∖Γ_*D*_. In order to ensure well‐posedness of the continuum model, so‐called *backflow stabilizations* may be added to Neumann boundaries, at the cost of perturbing imposed value of the boundary condition.^25^

An important aspect to mention is that in Equation (1) the pressure field is defined up to a time‐dependent spatial constant since ∇*p*(***x***, *t*) = ∇(*p*(***x***, *t*) + *δ*(*t*)). For pure Dirichlet problems (Γ_*D*_ = *∂*Ω), this constant remains undefined and should be fixed numerically. Neumann boundary conditions on a part of the boundary uniquely determine the pressure constant, but this value can be seen as arbitrarily user‐defined; indeed the velocity field depends only on the gradient of the pressure and not on its absolute value. This has the consequence that inverse problems based on the incompressible Navier–Stokes equations or some derived simplification, can only uniquely determine *a relative pressure field* with respect to some reference value, for any instant of time.

The arterial system takes an active part in continuously delivering blood at high pressure to the peripheral vasculature.^26^, ^27^ In particular, the large arteries deform elastically under increasing blood pressure during systole and act as a reservoir (“Windkessel”) storing blood which is ejected during diastole. Also the long muscular arteries and arterioles actively control the blood propagation to tissue and organs by different mechanisms.^26^, ^27^ In a mechanistic setting, the elastic deformation, as a response to forces exerted by the blood flow on the vessel wall, can be accounted for by coupling the Navier–Stokes Equations (1) with the equations of motion of (non)linear solid mechanics:

(3) $$\rho_{s}\frac{\partial^{2}}{\partial t^{2}}d_{s}-\nabla \cdot \sigma_{s}\left(d_{s},\theta_{s}\right)=0$$

with *ρ*_*s*_ the density of the solid tissue and $d_{s}:\Omega_{s}\times T\mapsto {\mathbb{R}}^{d}$ denoting the displacement vector of material points contained in the solid domain Ω_*s*_. The Cauchy stress tensor, ***σ***_*s*_, the distribution of stresses inside the material, depends, in the general case, nonlinearly not only on the displacement ***d***_*s*_ (and its derivatives), but also on parameters *θ*_*s*_ of a constitutive law characterizing the material.^28^ Biological tissues are often modeled as hyperelastic materials with phenomenologically derived constitutive laws. These models establish a nonlinear relationship between deformation and stresses via a strain‐energy function describing the material properties, which can include inhomogeneity and anisotropy, respecting, for example, the layered structure and fiber orientation of the arteries.^29^, ^30^ The equation of motion (3) requires initial conditions for the displacement, ***d***_*s*_(*t*_0_), and for the velocity field, $d_{s}\left(t_{0}\right)$. Boundary conditions in arterial models impose combinations of Dirichlet (***d***_*s*_ = ***g***_*s*,*D*_) and Neumann boundary conditions (***σ***_*s*_ ***n*** = ***g***_*s*,*N*_) in the radial and tangential directions, or Robin‐type conditions with elastic and viscoelastic coefficients *α*_*s*_ and *β*_*s*_ in order to represent the effect of the surrounding organs,^31^

$$\sigma_{s}n=-\alpha_{s}d_{s}-\beta_{s}d_{s}-p_{0}n,$$

where *p*_0_ is the external pressure.

### 2.2 Geometrically reduced‐order models

Full‐scale hemodynamic simulations of the complete vasculature will remain unfeasible for the foreseeable future.^32^ Therefore, detailed 3D computations are restricted to particular regions of interest of the cardiovascular system (for instance the aortic arch, heart valves, aneurysms or the carotid artery exhibit strong 3D flow phenomena) and have to be carried out within truncated computational domains. Geometrically reduced‐order models (GROM) can be derived with the aim to deliver realistic boundary conditions for full‐dimensional models, accounting for the effects of the omitted parts of the vascular system, or in order to construct a surrogate model of the full‐dimensional system, which can be solved more easily.

**FIGURE 1:**
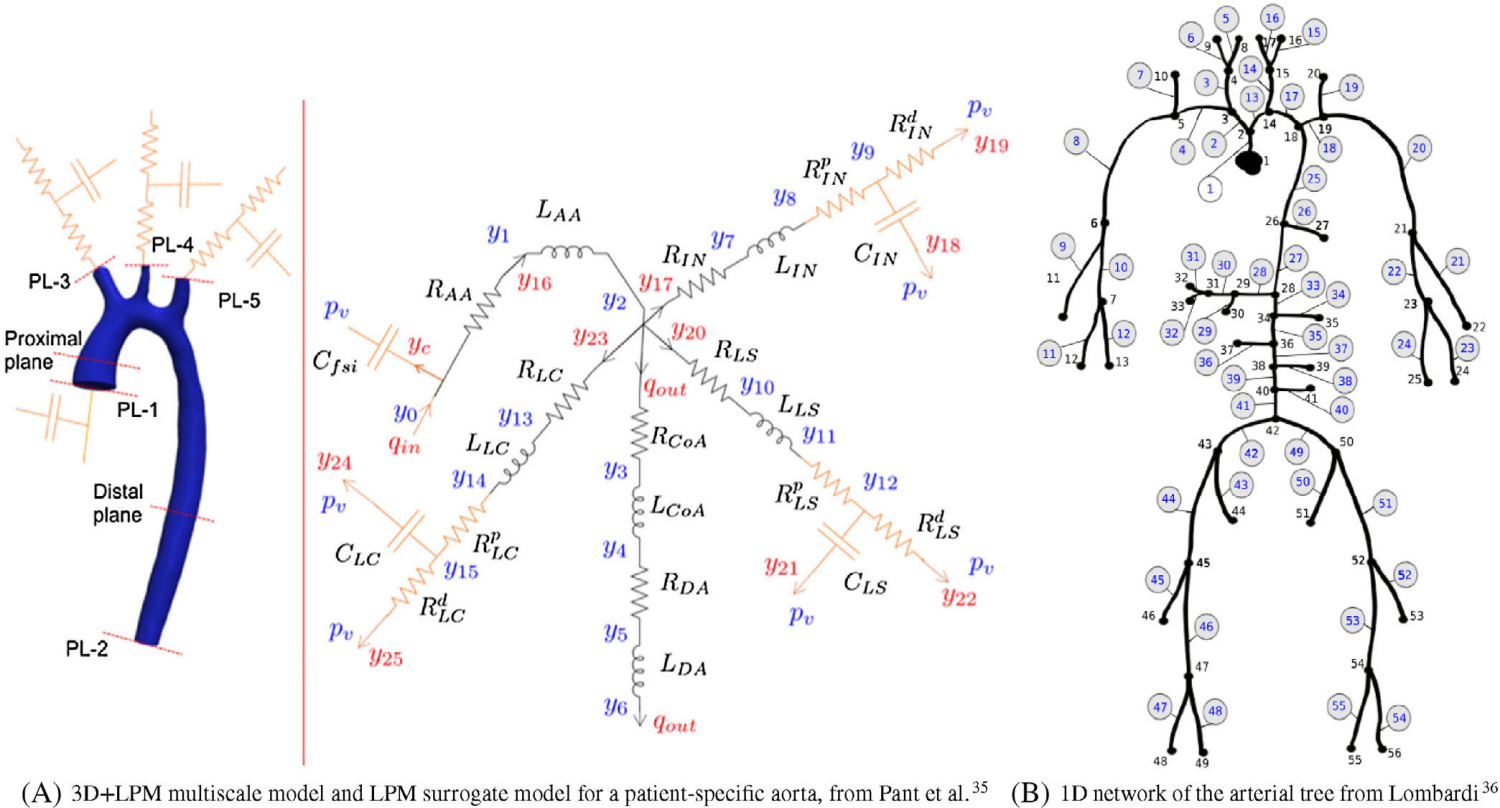
Examples of reduced‐order modeling approaches. (A) and multiscale 3D + LPM setting (left) and 0D surrogate LPM (right) for a patient‐specific aorta^33^ and (B) a complete 1D network of the arterial network.^34^ Reprinted with permission

Lumped‐parameter models (LPM) group the spatially distributed vascular system into the so‐called compartments, over which the conservation laws are averaged to obtain ordinary differential equations (ODE) modeling the bulk hemodynamics, for example, in terms of the flow rate and the pressure. LPM have been used to simulate the vascular system via multiple coupled compartments and to model the truncated vasculature, acting as a Neumann boundary condition for the higher dimensional models (see Figure 1A).

The most popular choice is the *three‐element Windkessel model*, including the vessel compliance *C*, the proximal resistance *R*_*p*_ and the distal resistance *R*_*d*_ ^35^ (proximal or distal refer to the interfaces of the modeled vessel situated nearer and farther from the heart). This model is given by the following ODE in terms of the distal pressure *p*_*d*_(*t*):

(4) $$\begin{array}{r} C\frac{dp_{d}}{dt}+\frac{p_{d}}{R_{d}}=Q,\\ R_{p}Q+p_{d}=P, \end{array}$$

where *P* and *Q* denote the pressure and flow rate at the proximal interface.

Modeling (parts of) the vascular system as a network of 1D compliant pipes, simplified equations of mass and momentum conservation can be derived, for example, in terms of the cross‐sectional area *A*(*x*, *t*) and the flow rate *Q*(*x*, *t*),

(5) $$\begin{array}{r} \frac{\partial A}{\partial t}+\frac{\partial Q}{\partial x}=0,\\ \frac{\partial Q}{\partial t}+\frac{\partial \;}{\partial x}\left(\alpha \frac{Q^{2}}{A}\right)+\frac{A}{\rho }\frac{\partial p}{\partial x}+k_{r}\frac{Q}{A}=0. \end{array}$$

The system is closed with a suitable constitutive law relating the internal pressure *p*(*x*, *t*) to the vessel wall mechanics,^32^ $p=\Phi \left(A;\dot{A},\ddot{A},\ldots \right)$. The constant *k*_*r*_ denotes the viscous resistance per tube unit length. Coupling conditions, imposing continuity of the fluxes and the total pressure need to be applied at bifurcations and discontinuities in the material properties. 1D vascular networks are often used as “standalone” surrogate models, as illustrated in Figure 1B, but sometimes coupled to full‐dimensional models.

The coupling of models of different geometric dimension is referred to as geometric multiscale modeling.^36^, ^37^ Typically, the coupling is done via

$$g_{N}=-Pn\;\mathrm{on}\;\Gamma ,\;Q=\int_{\Gamma }u\cdot n\mathrm{d\Gamma}$$

with ***n*** being the normal surface vector and Γ ⊂ *∂*Ω the part of the boundary where the coupling is enforced.

## 3 MEASUREMENTS OF BLOOD VESSELS AND FLOW

Acquisition of hemodynamic data is required in the current clinical practice to estimate quantities of diagnostic interest, and in addition serves as input for inverse problems. This section summarizes the different types of hemodynamic data and the corresponding measurement techniques.

### 3.1 Anatomy

The anatomy of the vessels is routinely used in the clinical assessment of vascular pathologies. Moreover, anatomical images provide—after segmentation and mesh generation using specialized software—the computational domain Ω used for the blood flow simulations and the inverse problems. Moreover, by segmenting, for example, the arterial wall over time one can measure a surrogate of the wall displacement ***d***_*s*_, which can be used in the context of inverse problems.

#### 3.1.1 Computerized tomography angiography

Computerized tomography (CT) measures X‐ray attenuations induced by different tissues inside the body. Multiple X‐ray measurements taken from different angles are reconstructed into a single tomographic image. In CT angiography (CTA), a contrast media (typically iodine‐based) is injected into the subject's bloodstream in order to distinguish the blood vessels from their surroundings.^38^ The main advantage of CTA is its high spatial resolution, which may be submillimetric depending on the size of the region if interest. CTA is usually obtained at only one cardiac phase, since for prolonged scan times, the radiation dose can exceed the annual recommended maximum level.^39^

**FIGURE 2:**
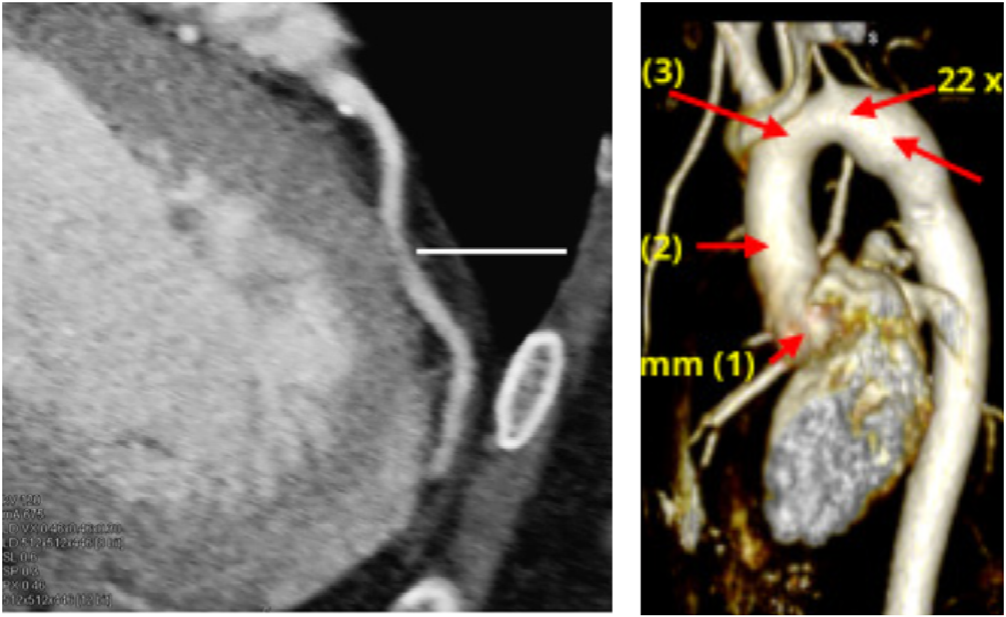
Anatomical images of blood vessels. Coronary arteries via CTA^40^ (left) and Coarctations of the aorta via MRA after thresholding non‐blood tissues^41^ (right). Reprinted with permission

However, in research settings cardiac‐gated CTA images are obtained allowing to obtain cardiac and arterial wall motion. An example of a CTA image of a patient's aorta is shown in Figure 2 (left column).

#### 3.1.2 Magnetic resonance angiography

Magnetic resonance imaging (MRI) functions by magnetically exciting hydrogen nuclei of the subject tissue and measuring the magnetization generated in response. From the characteristics of the measured signal, different types of tissues and their properties can be identified with great versatility. Manipulation of the sample by means of magnetic field gradients allows to encode, for example, spatial location, to create a 2D or 3D image, but also sensitizes the measurement to specific physical processes of interest, such as diffusion or flow.

Magnetic resonance angiography (MRA) is a variant of MRI for the context of anatomical vessel imaging, usually performed to extract the 3D blood vessel lumen, often using additional contrast media in order to alter the magnetic properties of the blood and to provide an improved contrast with respect to other tissues.^42^ An example of a MRA image of a patient's aorta is shown in Figure 2 (right column).

Due to its limited spatial resolution (around 2 mm voxel size), measuring the arterial wall dynamics is still not reliable: for instance, in large arteries deformations of the wall range between 2 mm to 4 mm, of the same order of the spatial resolution of MRA.^43^ Additionally, noise and other imaging artifacts severely corrupt the recovery of the arterial wall motion dynamics.

### 3.2 Blood flow velocity

Currently, the clinically relevant measurement techniques to assess hemodynamic flow velocities ***u*** are Doppler ultrasonography and Phase‐Contrast MRI (PC‐MRI). Both methods a non‐invasive.

#### 3.2.1 Doppler ultrasonography

**FIGURE 3:**
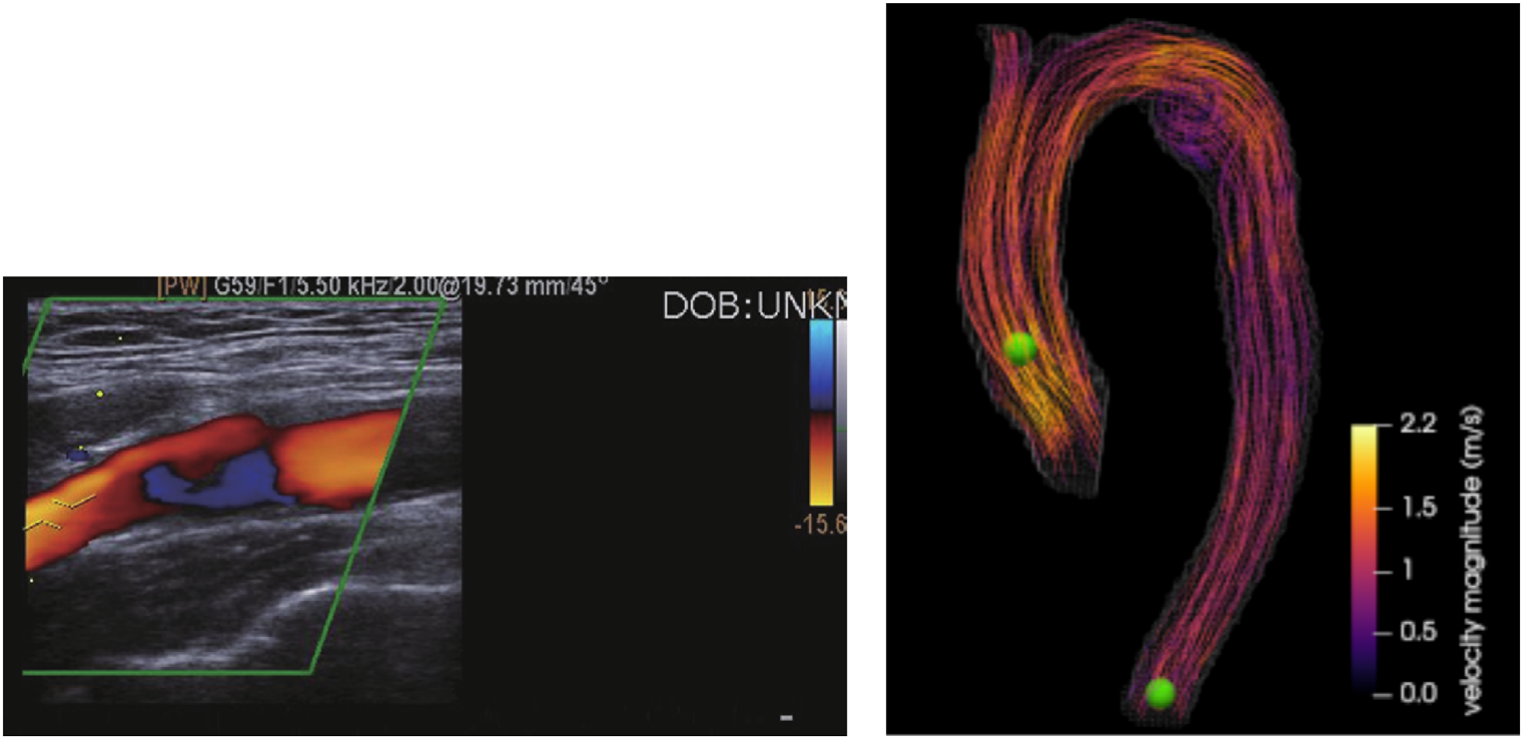
Blood flow velocity measurements. Doppler measurement of the internal carotid artery^45^ (left) and 4D Flow MRI of an CoA^41^ (right). Reprinted with permission

Doppler ultrasonography is capable of real‐time local velocity measurements along a beam or in two‐dimensional (2D) planes.^26^ It is versatile, non‐invasive, free of ionizing radiation and can detect relatively small structures, such as leaflets and narrow jets.^7^ Typical values of axial resolution range from 0.3 mm to 1.5 mm depending, for example, on the ultrasound frequency. The attenuation of the ultrasound beam increases at higher frequencies, so there is a tradeoff between penetration depth, axial spatial resolution and proximity to the probe.^44^ A further limitation of Doppler ultrasonography is that the maximum velocity that can be measured is limited within a certain range depending on the acquisition parameters. In that case, the velocity will be displayed opposite to the actual flow direction showing *velocity aliasing*.^44^ A typical 2D Doppler ultrasonography measurement is illustrated in Figure 3 for the carotid artery.

#### 3.2.2 Phase‐contrast magnetic resonance imaging

Information on the motion of a tissue—including blood flow—can be encoded with MRI in the phase of the signal emitted by the tissue by application of adequate magnetic gradients.^46^, ^47^ The procedure is known as *phase contrast MRI* (PC‐MRI). In time‐resolved measurements of vascular flow, images have to be acquired over multiple cardiac cycles, implicitly producing phase‐averaged data. Usually, 20–30 cardiac phases are considered.

2D PC‐MRI is a standard and widely available technique in clinics,^48^ with typical in‐plane resolutions of 1.5 mm to 2 mm and through‐plane resolutions of around 6 mm. The acquisition time corresponds to about one breathhold per 2D plane.

3D PC‐MRI—when time‐resolved, also called *4D Flow* ^49^—is usually acquired at a isotropic spatial resolution of 2 mm to 3.5 mm with scan durations between 10 and 20 minutes,^50^ therefore preventing its widespread clinical use. However, the acceleration of 4D Flow sequences is a very active research field.^51^, ^52^, ^53^ A further limitation of 4D Flow is that the maximum resolved velocity is determined by the scanner operator, larger velocities resulting in aliasing. This upper velocity limit, the so‐called *VENC*, relates to the strength and duration of the velocity encoding gradients.^49^ As a consequence, a larger *VENC* also leads to higher noise intensity and iterative adaptation may be required to find an optimal value.

Figure 3 (right column) displays an example of blood flow streamline visualization of in vivo 4D Flow data.

The 4D Flow measurement protocol can also be extended to measure turbulence statistics^54^, ^55^, ^56^, ^57^ (i.e., the Reynolds stress tensor^58^).

### 3.3 Pressure

As indicated in Section 2.1, pressure measurements are always relative with respect to a reference value, i.e., differences *p*(***x***_*a*_) − *p*(***x***_*b*_) between two locations ***x***_*a*_ and ***x***_*b*_.

In the clinical practice, both invasive and noninvasive approaches measure the difference between the patient's and the atmospheric pressure, making the values comparable among subjects.

#### 3.3.1 Sphygmomanometer (non‐invasive)

Monitoring the blood pressure non‐invasively may be the simplest and most widely used measurement to assess cardiovascular health. That measurement device—a sphygmomanometer—is often composed of an inflatable cuff to collapse and then release the artery in a controlled fashion and a device to measure the pressure applied.^59^ However, it is well known that sphygmomanometer pressure provides only an indication of the intra‐lumen blood pressure, and the relation between both depends on the compliance of the whole cuff‐skin‐arterial‐muscle system.^60^

#### 3.3.2 Applanation tonometry (non‐invasive)

Applanation tonometry flattens a small part of the eyeball under a tiny sensor to measure pulse pressure.^61^ It is nowadays widely used in clinical practice and research to measure arterial waveforms, in particular in the carotid and femoral arteries.^62^ Artifacts in the measurements are common, for example, due to respiration (especially in the carotid), and arterial motion requires the sensor to be stabilized by the operator.

#### 3.3.3 Catheterization (invasive)

As mentioned in the introduction, the functional relevance of an obstruction is usually characterized clinically by the pressure difference across it. Moreover, arterial stiffness calculations require pressure measurements—relative to the atmospheric pressure—to be comparable among patients.

The intra‐arterial spatial distribution of the blood pressure can be measured by means of catheterization.^4^ This technique consists in inserting a catheter equipped with a pressure transducer into the vasculature of the patient and maneuvering it, under local anesthesia and guided by fluoroscopy, to the location of interest. Although it is the “gold standard” for pressure quantification, the invasive nature of the method is associated with a risk of complications.^63^, ^64^, ^65^

**FIGURE 4:**
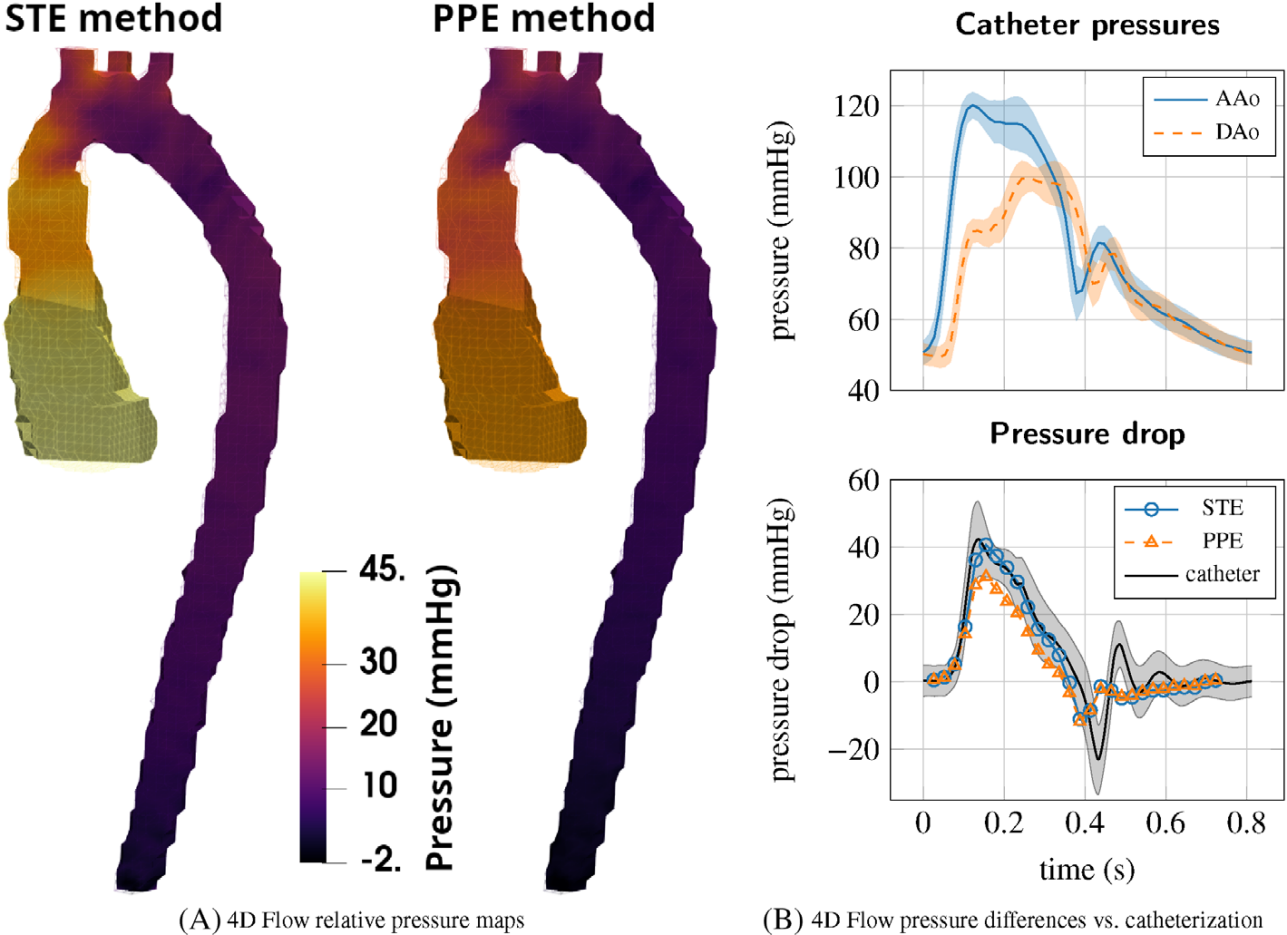
4D Flow‐based relative pressure estimation compared to catheter data, for a patient of CoA. (A) PPE and STE results at peak systole (pressure was arbitrarily set to zero at the end of the descending aorta [DAo]), (B) top: catheterization pressure measurements (with respect to atmosphere) in the ascending aorta (AAo) and DAo; bottom: DAo–AAo pressure difference from PPE/STE compared to catheterization. The shaded area indicates ±2 standard deviations of the catheterization pressures over cycles. ((b) adapted and reprinted with permission from Nolte et al.^41^)

The precision of the pressure measurements can be considered within a few mmHg. In the phantom experiments of Nolte et al.,^41^ the standard deviation of the subtraction of two pressure measurements was estimated to be around 5 mmHg, while in in vivo measurements it is expected to be twice as large.^66^ An example of in vivo catheter measurements in an aortic coarctation (CoA) patient is shown in Figure 4B, in two locations proximal and distal to the CoA, displaying also the measurement uncertainty between cycles. In addition to the measurement imprecisions, the presence of the catheters in the blood could also alter the flow and it is has been shown in in vitro and in silico studies that it could lead to possible overestimations in the pressure measurements.^67^

## 4 DIRECT PRESSURE GRADIENT COMPUTATION FROM VELOCITIES

We start this review article with the inverse problem most widely used in cardiology practice: pressure gradient estimation from velocity measurements. To justify the use of more advanced methods—based on 4D Flow—we first start with the simplest approaches based on the Bernoulli equation. It is important to remark that the direct methods reviewed in this section do not require solving optimization problems. Instead, they are formulated such that a pressure gradient estimate can be calculated directly (i.e., with a single linear system solution) from the available velocity data. The methods require the fluid density and viscosity, for which literature values are assumed.

### 4.1 Bernoulli‐based methods

Pressure gradient estimation using the Bernoulli equation is routinely used in the clinical practice since the required flow velocity measurements such as Doppler ultrasonography or 2D PC‐MRI are widely clinically available.^68^ Although they are mathematically trivial, we briefly describe the Bernoulli‐based approaches here for their practical relevance, in descending order of their data requirements.

When it is assumed that the pressure gradient is dominated by convective and inertial terms, the pressure difference between two points ***x***_*a*_ are ***x***_*b*_ along the flow trajectory ***y*** (*s*), *s* = [0, 1], is given by

(6) $$p\left(x_{a}\right)-p\left(x_{b}\right)\approx \frac{\rho }{2}\left({\left\|u\left(x_{b}\right)\right\|}^{2}-{\left\|u\left(x_{a}\right)\right\|}^{2}\right)+\rho \int_{0}^{1}\frac{\partial u}{\partial t}\cdot \frac{dy}{ds}\left(s\right)ds,$$

with ***y*** (0) = ***x***_*a*_ and ***y*** (1) = ***x***_*b*_. This method requires measurements along the trajectory but also knowledge of the trajectory itself, although some assumptions can be made on the latter.^69^ For those reasons, Equation (6) is not widely used in clinics but provides an interesting comparison to other methods when 4D Flow is available because of its reduced computational complexity.^70^

In the presence of a stenosis it is assumed that the pressure gradient ∇*p* is only dominated by the convective term − *ρ**u*** ·∇ ***u***. In this case it can be shown starting from Equation (1) that $p\left(x_{a}\right)-p\left(x_{b}\right)\approx \frac{\rho }{2}\left({\left\|u\left(x_{b}\right)\right\|}^{2}-{\left\|u\left(x_{a}\right)\right\|}^{2}\right)$. This computation requires measuring the velocity at two carefully chosen locations, for example, via two planes in 2D PC‐MRI, which may not be aligned with the flow directions. Furthermore, it is unknown which points in both planes correspond to the same streamline, hence simply the peak velocities are selected.

Due to the aforementioned drawbacks, a simpler version is used in the clinical practice: the simplified Bernoulli equation $p\left(x_{a}\right)-p\left(x_{b}\right)\approx \frac{\rho }{2}{\left\|u\left(x_{b}\right)\right\|}^{2}$, where ***x***_*b*_ is located in the jet exiting the obstruction and it is assumed that the velocity is much larger after the obstruction. Only one measurement is required of the jet velocity.

### 4.2 Navier–Stokes‐based methods

The assumption that the pressure gradient is dominated by the convective term is restrictive in compliant vessels and mild obstructions. Neither complex 3D flow patterns nor dynamic effects, shape of the obstruction, or turbulence are taken into account. As a consequence, Bernoulli methods are in many cases not appropriate to estimate realistic pressure gradients.

When information about the entire flow field in the region of interest is available, for example, 3D PC‐MRI data, more sophisticated methods may be used to reconstruct fields of the relative pressure in the entire domain. These methods require a 3D representation of the vessel lumen Ω, i.e., the computational domain, via segmentation of magnitude image or from an average of the velocity images.

The available methods can be grouped into two categories, computing (1) 3D maps of the relative pressure or (2) pressure differences between predefined locations. In both settings, the pressure constant needs to be fixed arbitrarily by specifying a reference pressure.

#### 4.2.1 3D Spatio‐temporal pressure maps

The classical approach to compute the pressure field *p*(***x***, *t*) from given velocity data ***u*** is by taking the divergence of (1), which results in the so‐called pressure Poisson Equation (PPE)^71^, ^72^, ^73^: find $p\left(\cdot ,t\right):\Omega \to \mathbb{R}$, such that

(7) $$-\Delta p=\nabla \cdot \left(\rho \frac{\partial u}{\partial t}+\rho \left(u\cdot \nabla \right)u-\mu \Delta u\right)=\nabla \cdot R\left(u\right)\;\text{in}\;\Omega .$$

Here, the assumption of blood to be a Newtonian fluid with dynamic viscosity *μ* was made and *R*(***u***) is shorthand notation for the velocity terms of the Navier–Stokes equation. Numerical solution of (7) requires boundary conditions for *p* on the entire domain boundary *∂*Ω. Taking the dot product of (1) with the outward unit normal vector ***n*** on *∂*Ω yields the Neumann boundary condition

(8) $$n\cdot \nabla p=-n\cdot R\left(u\right)\;\mathrm{on}\;\partial \Omega .$$

The solution of the Neumann boundary value problems (7) and (8) is defined up to a spatial constant *c*(*t*). The derivation of (7) invokes higher regularity assumptions for the pressure and the velocity, i.e., higher derivatives are required to exist for both *p* and ***u*** than originally in (1). As a consequence, the space of admissible functions among which the pressure is sought is a subset of the natural space.

This drawback can be circumvented by an approach proposed by Cayco and Nicolaides^74^ and adopted for the context of 3D PC‐MRI by Švihlová et al.,^75^ by introducing a divergence‐free auxiliary function $w\left(\cdot ,t\right):\Omega \to {\mathbb{R}}^{3}$, such that,

(9) $$\begin{array}{r} -\Delta w-\nabla p=R\left(u\right)\;\text{in}\;\Omega \\ \nabla \cdot w=0\;\text{in}\;\Omega \\ w=0\;\mathrm{on}\;\partial \Omega . \end{array}$$

Due to its resemblance to an inhomogeneous Stokes equation, the method is referred to as Stokes estimator (STE). Solving Equation (9), the pressure *p* is obtained together with the auxiliary function ***w***, whose purpose is to regularize the ill‐posed pressure gradient recovery problem. In the ideal scenario with perfect velocity measurements—*R*(***u***) being truly irrotational and no numerical errors—we have ***w*** = 0. Note that ***w*** could be used to explore the quality of the 4D flow data as proposed in.^76^

The PPE and STE methods were compared in numerical studies.^75^, ^77^ In Nolte et al.,^41^ the STE and PPE methods were evaluated using in vitro and in vivo MRI data in comparison to ground‐truth catheterization pressure measurements. These studies^41^, ^75^, ^77^ showed that the theoretical advantages of the STE method over the PPE method have a significant effect on the results in convection dominated flow regimes (elevated Reynolds numbers), as can be expected in clinically relevant severe stenoses. In addition, the latter study^41^ found an improved robustness to different segmentations of the STE with respect to PPE. Figure 4A shows STE and PPE relative pressure maps computed from 4D Flow data of a CoA patient. The larger pressure gradient obtained with the STE method is in closer agreement with catheterization data, as shown in Figure 4B.

While the original PPE method neglects the diffusive term in (7), a modification was recently proposed accounting for viscous effects.^27^ A theoretical analysis presented in Araya et al.^78^ indicated an improved accuracy compared to the standard method. The authors of the present paper performed preliminary computations on the phantom data of Nolte et al.^41^ including the viscous effects in the PPE, and the differences with the standard PPE appeared to be negligible. Further improvements to the treatment of the viscous effects were reported by Pacheco,^79^ where additionally the sensitivity of different treatments of the convective effects to noise in the data was studied.

#### 4.2.2 Pressure differences over predefined locations

Other approaches have been proposed for the computation of averaged pressure differences between two predefined cross‐sections of a blood vessel from 3D velocity data.

The first reported approach is the so called work‐energy relative pressure (WERP) method derived from the energy balance of Equation (1). Computationally, it only requires evaluating integrals of the data over the lumen of interest without solving a linear system.^80^ WERP showed increased accuracy with respect to the PPE,^80^ but less accurate than the STE.^77^ Bertoglio et al.^77^ also proved that WERP may have a considerable bias when noise in the data is present. A simplification of the WERP method was presented in Donati et al.^81^ (Simplified Advective WERP, or SAW) by taking only the boundary integral of the advective energy where the maximal velocity is present, therefore requiring only 2D PC‐MRI data.

The energy balance in WERP is obtained by taking the scalar product of (1) with the flow velocity itself. However, other functions could be used, satisfying some properties, for instance, the velocity of a Stokes flow in the same domain. The testing Stokes flow will depend on the chosen planes. Two variants of such approach have been proposed: the integral momentum relative pressure (IMRP) estimator^77^ and the virtual WERP (*v*WERP) method.^82^ The difference lies in the treatment of the convective terms. The *v*WERP method provided an increased accuracy compared to PPE and IMRP, with respect to catheter measurements in aortic flows,^82^ and obtained highly accurate estimates of the intraventricular pressure gradient using in silico data.^70^ Moreover, while the WERP method requires the vessel section of interest not to include any bifurcations (such as the supra‐aortic branches), this is not the case in the *v*WERP and IMRP methods. The *v*WERP was also recently applied to the estimation of pressure gradients in the brain vasculature,^83^ where it was found that the accuracy depends on the spatial image resolution in those small vessels. Another recent study used the *v*WERP for the clinical assessment of hemodynamics in dilated cardiomyopathy.^84^

#### 4.2.3 Inclusion of turbulent effects

Some of the pressure estimation methods haven been extended for turbulence by including measurements of the Reynolds' stresses (corresponding to the covariance of the turbulent velocity), basing the methods on the Reynolds‐averaged Navier–Stokes equations (RANS)^58^ rather than (1). RANS describe the evolution of the statistical mean flow, depending on the turbulent fluctuations in terms of the Reynolds' stresses. The methods differ from their non‐turbulent versions in that they include the Reynolds' stresses as obtained with the extended 4D Flow sequence, described in the previous section. Turbulence extensions were studied for the PPE method,^85^ and the WERP and vWERP methods.^86^ Marlevi et al.^86^ compared these methods for both in silico and in vivo data, showing the *v*WERP's extension (denoted *v*WERP‐t) to be the most accurate.

So far, no studies have been conducted extending the STE method by turbulence effects. Given measurements of the Reynolds' stresses, the method can be extended in straight‐forward manner, like the PPE method, by using the RANS equations instead of the Navier–Stokes equations.

### 4.3 Discussion and perspectives

Accuracy comparisons between STE and *v*WERP—the two best performing and validated methods so far—using in vitro or in vivo data sets have not yet been performed, in particular including Reynold's stress measurements. In terms of the method of choice, approaches computing pressure differences over predefined locations are computationally cheaper compared with computing pressure maps with PPE or STE methods. However, the former need to define the position and orientation of the planes in the lumen before the pressure difference computation, while the latter allows to explore the whole pressure field first.

One particularly attractive clinical application of 4D Flow based pressure map estimators is pediatric pathologies. In many of these diseases, the flows are very slow and therefore the circulation works at low pressures. In these cases, pressures are of order of a few mmHg but catheters have measurement errors of comparable magnitude. Moreover, the location of the maximal pressure gradient is unknown and therefore probing the whole pressure field from 4D Flow data could lead to more accurate diagnosis of the pathology.

A common measure in the clinical practice are the so‐called *peak‐to‐peak* pressure differences, which compare the difference in the pressure maxima registered at different locations during the complete cardiac cycle, thus taking into account time shifts due to the vessel elasticity. Usually, clinical reports of a patient's intra‐vessel pressure will be based on this quantity, and not the instantaneous pressure difference as computed by the approaches discussed in this section. An interesting and challenging research line, and of great clinical relevance, is the correct transfer from sphygmomanometer pressure measurements to blood lumen arterial pressures. This would allow fully non‐invasive peak‐to‐peak pressure measurements. In 4D flow‐based methods, the spatio‐temporal distributed data also allows to distinguish among the different contributions to the pressure gradient (inertial, advective and viscous) which could be explored clinically as markers of stenosis severity.

## 5 DATA ASSIMILATION METHODS

In this section, we will review modeling and numerical approaches that aim to estimate the following information of blood flow models by measurements of ***u***, *p*, ***d***_*s*_:

- Boundary conditions for the flow dynamics: (a) in−/outflow velocity profiles ***u***_*D*_, (b) the normal stress ***g***_*N*_, and/or (c) parameters of reduced‐order models *R*_*p*_, *R*_*d*_ and/or *C*.
- Elastic properties of the arterial walls *θ*_*s*_ and effects of surrounding organs *α*_*s*_.
- Compensating for errors in the initial condition $u\left(t_{0}\right),d_{s}\left(t_{0}\right),{\dot{d}}_{s}\left(t_{0}\right)$ and the computational geometry Ω.

This section will review the mathematical approaches which have been applied to perform these estimations. In the remainder of the article, the literature employing these methods in problems of blood flows will be reviewed.

Importantly, the optimized models lead to complete hemodynamic characterizations from partial (e.g., 2D) measurements. This is an important advantage over direct pressure estimation methods reviewed in Section 4.

### 5.1 General mathematical formulation

The model is a system of PDEs that describes the blood flow dynamics as discussed in Section 2. Let us introduce the following short‐hand notation for the space semi‐discrete numerical model, representing a differential algebraic equation (DAE),

(10) $$\dot{X}=A\left(X,\theta \right),$$

where $A:{\mathbb{R}}^{n}\times {\mathbb{R}}^{p}\mapsto {\mathbb{R}}^{n}$ is the model operator and $X\left(t\right)\in {\mathbb{R}}^{n}$, *t*_0_ ≤ *t* ≤ *T*, denotes the model state with *n* degrees of freedom and an initial condition *X*(*t*_0_). Depending on the effects represented in the model, it may include the fluid velocity, the solid displacements and velocities and/or the state variables of the reduced‐order models. The uncertain physical model parameters, such as constants pertaining to the boundary conditions or constitutive relations, are summarized in the parameter vector $\theta \in {\mathbb{R}}^{p}$. Parameters considered certain are implicitly included in the operator A.

A time discretization of the DAE (10) reads for the *n*th time step *t*_*n*_, *n* = 1, …, *N*, *t*_*N*_ = *T*, with the initial condition *X*_0_ = *X*(*t*_0_),

(11) $$X_{n}=A_{n}\left(X_{n-1},\theta \right).$$

We additionally assume that measurements, $Z_{n}\in {\mathbb{R}}^{m}$, are related to the state via the observation operator (possibly time‐dependent), ${ℋ}_{n}:{\mathbb{R}}^{n}\mapsto {\mathbb{R}}^{m}$, in a quasi‐static fashion such that

(12) $$Z_{n}={ℋ}_{n}\left(X_{n}\right)+\zeta_{n},$$

where $\zeta_{n}\in {\mathbb{R}}^{m}$ represents measurement errors, such as noise. This relationship allows *partial* measurements, or measurements of derived quantities of the state, to be used to estimate the state and/or model parameters.

We assume now that the measurement noise *ζ*_*n*_ follows a normal distribution with zero mean and a covariance matrix *W*_*n*_. It is further assumed a priori (i.e., without any prior knowledge on the measurements), that the knowledge on the initial condition *X*_0_ and the parameters *θ* is given by normal random variables with mean values ${\hat{X}}_{0}$, *θ*_0_ and covariances *C*_0_ and *P*_0_, respectively. Let us summarize the unknowns *X*_0_ and *θ* in the control vector, $\phi ≔\left(X_{0},\;\theta \right)$, and the corresponding initial guess and its covariance as $\phi_{0}≔\left({\hat{X}}_{0},\;\theta_{0}\right)$ and *Q*_0_: = diag(*C*_0_, *P*_0_). The adoption of a *Bayesian estimation approach*—i.e., maximizing the probability that the model observes the data given prior information—defines the following cost function weighting the discrepancy between the model state and the observations and the prior knowledge:

(13) $$J\left(\phi \right)=\sum_{n=0}^{N}{\left\|Z_{n}-{ℋ}_{n}\left(X_{n}\right)\right\|}_{W_{n}^{-1}}^{2}+{\left\|\phi -\phi_{0}\right\|}_{Q_{0}^{-1}}^{2}.$$

Evaluation of *J* in a particular value of *ϕ* implies solving (11) to obtain *X*_*n*_ for the given *ϕ*.

Data assimilation seeks the optimal trajectory of the state *X*_*n*_ by solving the minimization problem

(14) $$\underset{\phi }{\arg\;\min}\;J\left(\phi \right).$$

The solution process of Problem (14) is facilitated by the second term in the cost function, usually referred to as *Tikhonov regularization*. Imposing additional desired properties on the solution—for instance, low energy, smoothness or, as in (13), prior knowledge—regularization is crucial when problems are ill‐posed (e.g., noisy data and/or nonlinear models).

There are two main families of approaches to solve Problem (14): variational or sequential data assimilation. Both approaches have been applied to inverse problems in hemodynamics and shall be outlined in the following sections.

### 5.2 Variational data assimilation

Efficient solution of the minimization problem (14) with, for example, quasi‐Newton optimization algorithms, requires the gradient of the cost function *J* with respect to the control variables *ϕ*. Explicit approximation of the gradient with finite differences is excessively expensive in practice, since it scales linearly with the dimension of the control variables. In contrast, the *adjoint method* provides an elegant and efficient method to compute the gradient of *J*,^87^, ^88^ at a cost basically independent of the dimension of the control space.^89^

For the purpose of illustrating the adjoint method, we rewrite the PDE‐constraint (10) in an abstract form.^90^

(15) $$F\left(X,\phi \right)=0,$$

where *ϕ* represents all controls and the state *X* can be seen as the block‐structured vector containing the unknowns at all times (i.e., *X* = [*X*_1_, *X*_2_,ĳ,*X*_*N*_]). The boundary conditions are assumed to be included in *F*. We seek the gradient of *J* with respect to *ϕ*, in order to minimize the cost function *J* with constraint (15). The gradient, applying the chain rule, reads

(16) $$\frac{dJ}{d\phi }=\frac{\partial J}{\partial X}\frac{dX}{d\phi }+\frac{\partial J}{\partial \phi },$$

where partial derivatives $\frac{\partial J}{\partial X}$ and $\frac{\partial J}{\partial \phi }$ are easily obtained analytically. In contrast, computation of the so‐called sensitivities $\frac{dX}{d\phi }$, for example, by finite difference approximations, is extremely costly and generally impractical. Differentiating the model (15), an expression can be found for the sensitivity (assuming $\frac{\partial F}{\partial X}$ is invertible),

(17) $$\frac{\partial F}{\partial X}\frac{dX}{d\phi }+\frac{\partial F}{\partial \phi }=0\;\Leftrightarrow \;\frac{dX}{d\phi }=-{\left(\frac{\partial F}{\partial X}\right)}^{-1}\frac{\partial F}{\partial \phi },$$

where, again, the partial derivatives of *F* can usually be determined analytically in straight forward manner, and where $\frac{\partial F}{\partial X}$ represents the Jacobian of the forward model. Substituting (17) in (16) yields

$$\frac{dJ}{d\phi }=-\frac{\partial J}{\partial X}{\left(\frac{\partial F}{\partial X}\right)}^{-1}\frac{\partial F}{\partial \phi }+\frac{\partial J}{\partial \phi },$$

or, equivalently,

(18) $$\frac{dJ}{d\phi }=\lambda^{T}\frac{\partial F}{\partial \phi }+\frac{\partial J}{\partial \phi },$$

introducing the adjoint state, *λ*, satisfying

(19) $${\left(\frac{\partial F}{\partial X}\right)}^{T}\lambda =-{\left(\frac{\partial J}{\partial X}\right)}^{T}.$$

Equation (19) is the adjoint equation of the PDE‐constrained optimization problem. Accordingly, the gradient $\frac{dJ}{d\phi }$ is determined by solving the adjoint Equation (19) which depends on the forward solution *X*, and easy to compute partial derivatives of the model and cost function. A typical adjoint‐based optimization procedure is given in Algorithm 1.

ALGORITHM 1 **General adjoint‐based optimization algorithm**

Given an initial guess *ϕ* ^0^, repeat for *k* = 0,ĳ, *N*_max_, until convergence:

1. Compute forward solution *X* ^*k*^ for controls *ϕ* ^*k*^ [Equation (10)]
2. Compute adjoint solution *λ* ^*k*^ [Equation (19)]
3. Compute gradient $\left.\frac{dJ}{d\phi }\right|-0.0001{\mathrm{pt}}_{\phi^{k}}$ from *λ* ^*k*^ [Equation (16)]
4. Decrease the value of *J* using direction $\left.\frac{dJ}{d\phi }\right|-0.0001{\mathrm{pt}}_{\phi^{k}}$ (e.g., using some quasi‐Newton method) to obtain new controls ${\hat{\phi }}^{k}$
5. Set $\phi^{k+1}≔{\hat{\phi }}^{k+1}$

The structure of the adjoint equation corresponds to a linearization of the forward problem. Its linearity renders the solution relatively simple, with a cost comparable to or less than that of the forward problem. For time‐dependent problems, however, the adjoint Equation (19) runs *backward in time*, starting with a terminal condition at *t* = *T*, to *t*_0_. At every time step, the corresponding forward solution is required, hence its entire trajectory needs to be stored. This fact leads to substantial memory requirements which are often prohibitive in practice. A reduction in memory requirements at the cost of increased computation time is achieved by splitting the time interval into subintervals and storing forward solution checkpoints only at the start of every interval. The adjoint computation proceeds subinterval by subinterval, recomputing the forward trajectory from the checkpoints when needed, and discarding old checkpoints when these become unnecessary. Checkpointing schedules^91^ determine the optimal number of checkpoints in order to balance the tradeoff between storage needs and computation time according to the available resources and requirements.

Practical implementations have to choose if the adjoint equation is derived analytically, prior to discretization, or if a discrete adjoint is constructed from the discretized forward problem. The former approach is referred to as *optimize‐then‐discretize* (OD), the latter as *discretize‐then‐optimize* (DO). Both approaches have advantages and are employed successfully in practice (see, for example, the discussion in Gunzburger^92^). For instance, continuous adjoint equations (and cost function gradient) allow to use different and specific computational facilitators (e.g., stabilization schemes) for the forward and adjoint problems. However, gradients obtained with the OD approach are inconsistent with the discretized cost function and forward problem, which can cause optimization algorithms to fail. The DO approach avoids this issue and furthermore allows the adjoint to be derived by means of automatic/algorithmic differentiation tools (with their own benefits and pitfalls),^90^, ^93^ and is often preferred in practice. In general, adjoint‐based variational approaches are popular when the dimension of the control space is large, as is the case when estimating distributed boundary conditions and/or the initial conditions.

### 5.3 Sequential data assimilation

The adjoint‐based variational data assimilation approach fits the entire trajectory of *X*_*n*_ to the ensemble of observations. Sequential DA instead uses a recursive procedure in which all observations are assimilated during one forward time integration of the model, once they are ˝encountered.˝ Specifically, at each time *t*_*k*_ sequential methods find the estimate based on the cost function (13) with the upper limit of the sum set to the current iteration, *N* = *k*. In this way, at each assimilation instant, the new information is added to the previous ‘knowledge’ to improve the model predictions. In contrast to adjoint variational DA, the sequential approach considers at a time *t*_*k*_ all previously gathered observations, but not ‘future’ observations.

#### 5.3.1 Kalman filtering

Kalman filtering is the classic approach of sequential data assimilation^94^ and consists in an a priori prediction step (or forecast in DA jargon) by the numerical model and an a posteriori correction step (or analysis), incorporating the observations into the state estimation. For its introduction, let us consider a linear model for the discrete problem (11) with operator *A*_*n*_ at time *t*_*n*_ for an unknown true state $X_{n}^{t}$ to be estimated:

(20) $$X_{n}^{t}=A_{n}X_{n-1}^{t}+B_{n}.$$

*B*_*n*_ is the vector containing boundary condition and source terms. For simplicity, we assume that unknown parameters *θ* are appended to the state vector *X*_*n*_, and that *A*_*n*_ is extended accordingly by an identity matrix block, such that a pseudo‐propagation law *θ*_*n*_ = *θ*_*n*−1_ is obtained. We introduce an a priori prediction *X*_*n*_ of the unknown true state $X_{n}^{t}$ and an a posteriori correction ${\hat{X}}_{n}$, the estimation errors $\epsilon_{n}=X_{n}-X_{n}^{t}$ and ${\hat{\epsilon }}_{n}={\hat{X}}_{n}-X_{n}^{t}$, and the corresponding error covariance matrices,

$$P_{n}=E\left[\epsilon_{n}\epsilon_{n}^{T}\right],\;{\hat{P}}_{n}=E\left[{\hat{\epsilon }}_{n}{\hat{\epsilon }}_{n}^{T}\right].$$

Given a correction ${\hat{X}}_{n-1}$ at time *t*_*n*−1_, the a priori prediction at the next time step *t*_*n*_ is computed by propagating the correction with the forward model,

(21) $$X_{n}=A_{n}{\hat{X}}_{n-1}+B_{n}.$$

The Kalman filter finds the optimal a posteriori correction by balancing the model prediction with the observation data,

(22) $${\hat{X}}_{n}=X_{n}+K_{n}\left(Z_{n}-H_{n}X_{n}\right),$$

where *H*_*n*_ is the observation operator, also assumed linear, *Z*_*n*_ denotes the measurements. The weighting between model predictions and observations, *K*_*n*_, referred to as the Kalman gain, is defined such that the a posteriori error estimate variances ($\mathrm{tr}{\hat{P}}_{n}$) are minimized,

(23) $$K_{n}=P_{n}H_{n}^{T}{\left(W_{n}+H_{n}P_{n}H_{n}^{T}\right)}^{-1},$$

where *W*_*n*_ denotes the covariance matrix of the measurement error. For the covariance matrices, the prediction step is given by the recursion formula

(24) $$P_{n}=A_{n}{\hat{P}}_{n-1}A_{n}^{T},$$

with ${\hat{P}}_{0}$ a given initial condition. The correction error covariance is computed with

(25) $${\hat{P}}_{n}=\left(I-K_{n}H_{n}\right)P_{n}.$$

Summarizing, a time step of the Kalman filter computes in a prediction step priors of the state estimate and the covariance matrix (21), (24) followed by a correction step consisting in the computation of the Kalman gain (23) and updates of the state estimate and covariance (22), (25). For a complete and instructive derivation, the interested reader is referred to Asch et al.^95^

In the linear case, the Kalman gain contains the inverse of the Hessian of the cost function, and therefore provides an exact approach to solve the minimization problem but in a recursive fashion over time. In the nonlinear case, different approaches for computing *K*_*n*_ give rise to variants of the Kalman filter. The Extended Kalman filter is the direct adaptation of the linear version to nonlinear problems by linearization. It is impractical for most problems, since it requires a tangent linear model, operates on the full, dense covariance matrix and requires the nonlinearities to be weak. The Ensemble Kalman Filter^96^ (EnKF) and its variations^97^ and the Unscented Kalman Filter^98^ (UKF) use instead ensembles of state perturbations (referred to as ˝particles˝) to approximate the error statistics (state estimate mean and covariance). They differ mainly by using deterministic or stochastic particles, respectively, and the latter employing a low‐rank approximation of the covariance matrix, thus reducing the number of required particles.

Practical advantages with respect to variational DA are that storage of the state is not required for all times and that gradients of the cost function *J* are approximated using ˝derivative‐free˝ approaches. The price to pay is that the Kalman gain matrix *K*_*n*_—of the size of the dimension of the uncertain parameters and/or initial condition—is not sparse. Therefore, using Kalman filter methods for estimating *X*(*t*_0_) in realistic hemodynamic problems is computationally expensive if no assumptions are made to severely reduce the problem size. Also, the large number of particles required (for instance, 50–100 for the EnKF)^99^ results in a high demand in CPU time, since for each particle one independent forward problem has to be solved. These particle forward problems can be solved simultaneously on a parallel computer if enough computational resources are available.

#### 5.3.2 Reduced‐order Kalman filtering (perfect knowledge on *X*(*t*_0_))

Data assimilation problems can be greatly simplified by neglecting the uncertainty in the initial condition of the state (number of unknowns of the order 10^5^ to 10^7^) and only considering uncertainties in the parameters (typically dozens or less), describing, i.e., boundary conditions and material properties. Both variational and sequential DA methods are applicable to the resulting parameter estimation problem. For small numbers of parameters, the sequential approach offers the advantages of computational efficiency due to the recursivity avoiding the storage of the state, but also due to its implementational simplicity without the need of deriving or implementing adjoints.

A reduced‐order version of the UKF for parameter estimation was presented in Moireau and Chapelle,^100^ referred to as the reduced‐order unscented Kalman filter (ROUKF). It assumes that the uncertainties at the initial time are of low rank, for instance concentrated on the physical parameter *θ* to be estimated. Hence, the number of particles it employs is the number of parameters to be estimated plus one, which renders the problem very tractable since the solutions of the particles can be fully parallelized and the matrices involved in the Kalman filtering approach remain low rank. This approach has been extensively applied in blood flows as will be discussed in the following sections. The ROUKF method is presented in detail in Algorithm 2.

ALGORITHM 2 **Factorized Reduced‐order Unscented Kalman Filter (ROUKF) after Moireau and Chapelle.^100^**

**Problem:** Consider a parameter estimation problem (11)–(14) with nonlinear model and observation operators. The initial condition ${\hat{X}}_{0}\in {\mathbb{R}}^{r}$ is known exactly and *p* unknown model parameters $\theta \in {\mathbb{R}}^{p}$ are sought for which an initial guess ${\hat{\theta }}_{0}$ and, as a measure of its uncertainty, the covariance $P_{0}\in {\mathbb{R}}^{p\times p}$ are given.
**Definitions:** *X*_*n*_ denotes a prior prediction of the state and ${\hat{X}}_{n}$ a posterior correction. Let [*Y* ^(*)^] denote the matrix with the column‐wise collection of vectors $Y^{\left(1\right)},Y^{\left(2\right)},\ldots$. Define the *simplex sigma‐points* $I^{\left(i\right)},\ldots ,I^{\left(p+1\right)}\in {\mathbb{R}}^{p}$ given such that $\left[I^{\left(*\right)}\right]\equiv \left[I_{p}^{\left(*\right)}\right]\in {\mathbb{R}}^{p\times \left(p+1\right)}$ is computed recursively as^101^, ^102^

$$\left[I_{1}^{\left(*\right)}\right]=\left[-\frac{1}{\sqrt{2\alpha }}\;\frac{1}{\sqrt{2\alpha }}\right]\;,\;\alpha =\frac{1}{p+1},$$

and

$$\left[I_{d}^{\left(*\right)}\right]=\left[\begin{array}{cccc} \; & \; & \; & 0\\ \; & \left[I_{d-1}^{*}\right] & \; & \vdots \\ \; & \; & \; & 0\\ \frac{1}{\sqrt{\mathrm{\alpha d}\left(d+1\right)}} & \cdots & \frac{1}{\sqrt{\mathrm{\alpha d}\left(d+1\right)}} & \frac{-d}{\sqrt{\mathrm{\alpha d}\left(d+1\right)}} \end{array}\right]\;,\;d=2,\ldots ,p\;.$$

**Initialization:** initialize the sensitivities as

(26) $$L_{0}^{\theta }=\sqrt{P_{0}^{-1}}\;\left(\text{Cholesky factor}\right),\;L_{0}^{X}=0\in {\mathbb{R}}^{r\times p},\;U_{0}=P_{\alpha }\equiv \alpha \left[I^{\left(*\right)}\right]{\left[I^{\left(*\right)}\right]}^{T}$$

Then compute for n=1,…,N:

1. **Sampling:** generate *p* + 1 particles from the current state and parameter estimates, for *i* = 1, …, *p* + 1:

(27) $$\left\{\begin{array}{l} X_{n-1}^{\left(i\right)}={\hat{X}}_{n-1}+L_{n-1}^{X}C_{n-1}^{T}I^{\left(i\right)},\\ \theta_{n-1}^{\left(i\right)}={\hat{\theta }}_{n-1}+L_{n-1}^{\theta }C_{n-1}^{T}I^{\left(i\right)} \end{array}\right.$$

with *C*_*n*−1_ the Cholesky factor of $U_{n-1}^{-1}$.

2 **Prediction:** propagate each particle with the forward model and compute an a priori state prediction:

(28) $$\left\{\begin{array}{l} X_{n}^{\left(i\right)}=A_{n}\left(X_{n-1}^{\left(i\right)},{\hat{\theta }}_{n-1}^{\left(i\right)}\right),\;\theta_{n}^{\left(i\right)}=\theta_{n-1}^{\left(i\right)},\;i=1,\ldots ,p+1\\ X_{n}=E_{\alpha }\left(\left[X_{n}^{\left(*\right)}\right]\right)\equiv \alpha \sum_{i=1}^{p+1}X_{n}^{\left(i\right)},\;\theta_{n}={\hat{\theta }}_{n-1} \end{array}\right.$$

3 **Correction:** compute a posteriori estimates based on measurements for state and parameters:

(29) $$\left\{\begin{array}{l} \Gamma_{n}^{\left(i\right)}=Z_{n}-ℋ\left(X_{n}^{\left(i\right)}\right)\;i=1,\ldots ,p+1\\ L_{n}^{X}=\alpha \left[X_{n}^{\left(*\right)}\right]{\left[I^{\left(*\right)}\right]}^{T}\\ L_{n}^{\theta }=\alpha \left[\theta_{n}^{\left(*\right)}\right]{\left[I^{\left(*\right)}\right]}^{T}\\ L_{n}^{\Gamma }=\alpha \left[\Gamma_{n}^{\left(*\right)}\right]{\left[I^{\left(*\right)}\right]}^{T}\\ U_{n}=P_{\alpha }+{\left(L_{n}^{\Gamma }\right)}^{T}W_{n}^{-1}L_{n}^{\Gamma }\\ {\hat{X}}_{n}=X_{n}-L_{n}^{X}U_{n}^{-1}{\left(L_{n}^{\Gamma }\right)}^{T}W_{n}^{-1}E_{\alpha }\left(\left[\Gamma_{n}^{\left(*\right)}\right]\right)\\ {\hat{\theta }}_{n}=\theta_{n}-L_{n}^{\theta }U_{n}^{-1}{\left(L_{n}^{\Gamma }\right)}^{T}W_{n}^{-1}E_{\alpha }\left(\left[\Gamma_{n}^{\left(*\right)}\right]\right) \end{array}\right.$$

Note: $P_{n}=L_{n}^{\theta }U_{n}^{-1}{\left(L_{n}^{\theta }\right)}^{T}$ is an estimate of the parameter error covariance. *W*_*n*_ is the measurement error covariance matrix, i.e., for Gaussian white noise with fixed variance, *W*_*n*_ = *W* = *σ* ^2^.

State observers (perfect knowledge on *θ*)

When dealing with uncertainties in the initial condition only, alternative tractable filtering approaches can be formulated by modifying the system dynamics as:^103^

(30) $$\dot{\hat{X}}=A\left(\hat{X},\theta \right)+ℒ\left(Z-ℋ\left(\hat{X}\right)\right),\;\hat{X}\left(t_{0}\right)\neq X\left(t_{0}\right),$$

with ℒ a sparse operator. In practice, ℒ has to be designed for each type of measurements (e.g., displacements, velocities and/or pressures) and physics (e.g., fluid, solid or fluid–solid coupling) such that $\hat{X}\left(t\right)\to X\left(t\right)$ when t→∞, where *X*(*t*) denotes the true state.

This methodology is effective for estimating the state in presence of uncertainties in the initial guess but estimating model parameters is not possible. However, sequential data assimilation methods for parameter estimation can be combined with state observers in order to enable computationally inexpensive joint state/parameter estimation.^104^

### 5.4 Root‐finding formulation

Root‐finding is an approach for parameter estimation problems with measurements that are not distributed over the time interval, but either time‐averages of hemodynamic quantities or, for example, terminal target values. Instead of least‐squares minimization of the model–observation discrepancy using the quadratic cost‐function (13), the root‐finding problem reads: *find θ such that*

(31) $$Z-ℋ\left(X\left(\theta \right)\right)=0\;\text{with}\;X,\;\theta \;\text{satisfying}\;\left(10\right),$$

assuming that the measurements *Z* and $ℋ\left(X\right)$ correspond to a specified instant of time or cycle averages (and not temporally resolved data).

Such problems are usually solved by means of (quasi‐)Newton methods, requiring the Jacobian (or an approximation) with respect to *θ* of the residual $Z-ℋ\left(X\right)$ at every iteration of the algorithm. Due to the nature of the root‐finding problem, the number of measurements has to match the number of parameters for the roots of (31) to be determined, in contrast to least‐squares optimization approaches. Root‐finding lacks the optimality properties, the theory and generality of optimization, but can be very cheap in the specific cases where the method is applicable and converges. Details of the application of these methods to blood flows are given in Section 7.5.

## 6 ESTIMATION OF BOUNDARY CONDITION DATA

### 6.1 Preliminaries

Full‐dimensional hemodynamic simulations require inflow and outflow boundary conditions at the interfaces of the computational domain, accounting for the truncated vasculature. One of the most explored research topics in the field has been the estimation of distributed boundary conditions—i.e., velocity ***u***_*D*_ or normal stress boundary data ***g***_*N*_—for Problem (1) from distributed interior velocity measurements (e.g., from 2D or 3D PC‐MRI) using (mainly variational) data assimilation approaches. For the sake of readability, we structure this section by classifying the literature by stationary and time‐dependent settings.

### 6.2 Averaged boundary data

Some of the first inverse problems in blood flows were motivated by the limited clinical availability of measurements, which consisted mainly in flow rates and mean pressures. Imposing these types of data as boundary conditions was called *defective boundary conditions* and received significant attention in the first decade of this century. This “data imposition” involves solving an inverse problem.

Formaggia et al.^105^ treated the problem of imposing average pressure and flow rate boundary data in rigid‐wall flow problems. An inverse problem was formulated with the desired boundary data, i.e., either pressure or flow rate, as “measurement” while the other quantity was used as control variable. It was proposed to solve this problem using iterative minimization algorithms with the gradient of the cost function based on adjoint equations. Additional constraints on the boundary velocity were imposed. Existence of solutions is proved for the stationary linearized case, as well as for the Navier–Stokes problem with large viscosity. The problem for the transient case was formulated in a quasi‐stationary fashion for each time step. Numerical examples in transient 2D flows with one and two control boundaries exemplified the methodology, and the convergence of the numerical algorithm was proved. For the examples shown only a couple of iterations of the minimization procedure were needed. However, the convergence behavior in the presence of a larger number of control boundaries was not investigated.

Formaggia et al.^106^ developed the extension to fluid–solid interaction for the flow rate data case, where various numerical algorithms were proposed and investigated. However, for a similar 2D test case with two boundaries, the number of iterations increased by a factor of 10 with respect to the rigid walls example previously reported.^105^

### 6.3 Distributed boundary data in stationary flows

#### 6.3.1 Estimation of boundary normal stress

Distributed boundary normal stress ***g***_*N*_ were first estimated in D'Elia and Veneziani^107^ from synthetic velocity measurements using variational data assimilation on a Stokes model, which was later extended to the Oseen and Navier–Stokes equations in D'Elia et al.^108^ In both works, the optimal control problem was solved via the discretize‐then‐optimize approach and tested in 2D synthetic examples, with velocity measurements on interior slices. Conditions for the existence of a unique minimizer of the optimization problem were investigated, revealing that a Tikhonov regularization for the flow velocity allows to avoid constraints on the position of the measured data. Furthermore, it was shown that interpolating the data to different locations also had a regularizing effect, such that the Tikhonov regularization could be omitted.

Adib et al.^109^ addressed the problem of estimating boundary normal stress—assumed constant over each outlet boundary—by means of bruteforce minimization. Results computed for real 4D Flow data from patients with intracranial aneurysm were shown, where agreements between the optimized model and data were satisfactory even if data coming from a time‐dependent regime. However, the bruteforce optimization approach used scaled exponentially with the number of boundaries, so the authors recognized that this could be improved, for example, by introducing local minimization strategy.

A monolithic approach, considering the coupled state‐adjoint optimality system, was formulated by Zainib et al.^110^ to estimate distributed normal stress boundary conditions from 4D Flow‐type measurements. The main contribution of the work was to show that a reduction of the state‐adjoint optimality system dimension of more than three orders of magnitude is possible using proper orthogonal decomposition (POD).

#### 6.3.2 Estimation of boundary velocity profiles

First in Tiago et al.^111^ and Guerra et al.,^112^ the velocity boundary profiles ***u***_*D*_ of 2D fluid flow problems were optimized using a discretize‐then‐optimize approach, including a regularizer on the gradient of the boundary velocity. Later, the analysis was extended to 3D, allowing to recover helical and secondary flow patterns downstream of the inlet.^113^, ^114^ Numerical results showed that the regularizer allows to estimate ***u***_*D*_ from flow velocity measurements only in a subset of the domain. The existence of a minimizer of the optimization problem for the case that the velocity is measured everywhere in the domain was proven in a previous publication.^115^

Koltukluoğlu and Blanco^116^ first applied a similar problem formulation to experimental MRI phantom data. A optimize‐then‐discretize approach was adopted for the optimization problem. Due to the “full field” nature of the measurements, the optimized model's solution turned out to be a filtered, denoised version of the measurements.

### 6.4 Time‐dependent flow

#### 6.4.1 Estimation of boundary data only

**FIGURE 5:**
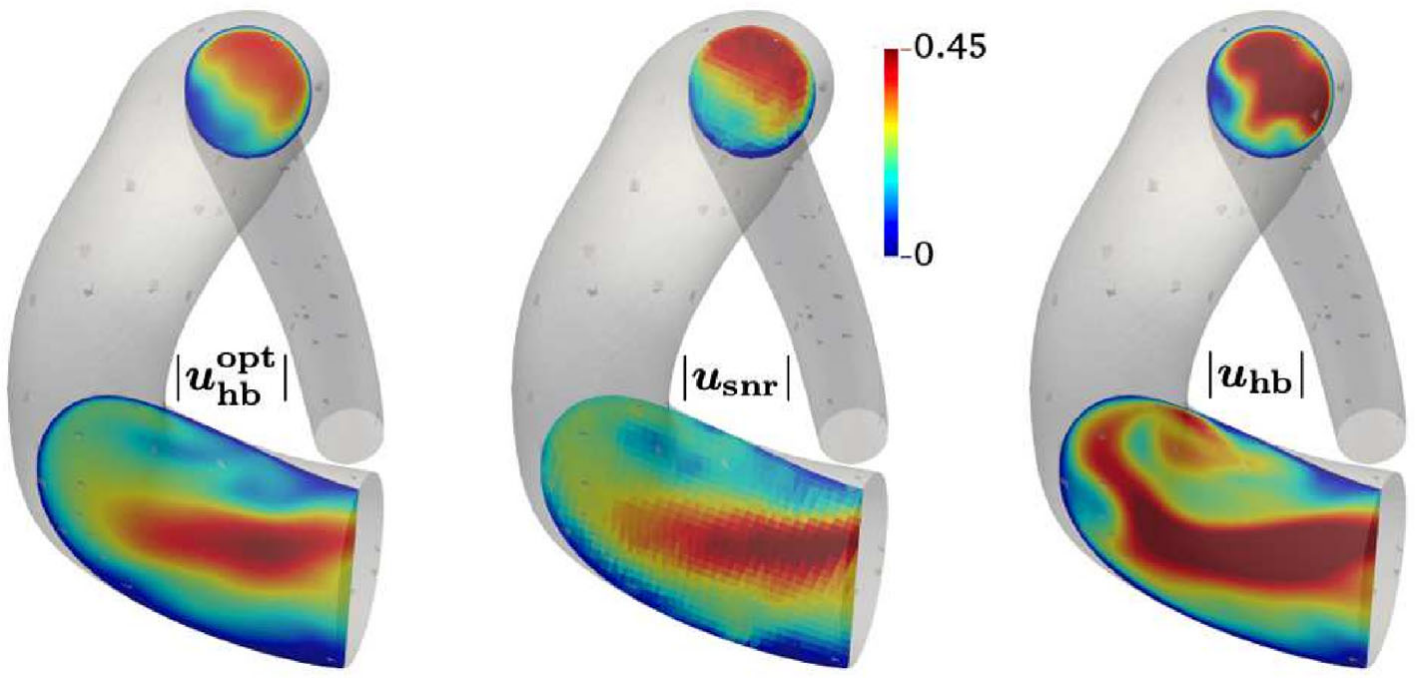
Velocity magnitude estimation applying spectral data assimilation to 4D Flow data of an aortic phantom.^117^ Left: optimized harmonic balance model; middle: 4D Flow data; right: harmonic balance forward simulation. Permission to be requested from Springer upon acceptance

In order to enable treating the 3D time‐dependent problem, Koltukluoğlu^117^ proposed to reduce the complexity of the inverse problem of velocity boundary data estimation from velocity data. At the core of this work was the assumption of a time periodic flow (reasonable for physiological blood flows) which allowed to address the problem in the frequency domain by means of an harmonic balance approach. Thus, the time‐dependent problem is rewritten as a sequence of coupled stationary estimation problems for each frequency. The approach avoids the estimation of the initial condition, considerably reducing the dimension of the optimization problem and making it tractable for adjoint‐based variational data assimilation. A further reduction of the size of the parameter space is achieved by selecting a sufficiently small number of frequencies (much smaller than the number of time steps). The method was assessed on real phantom 4D Flow MRI data, see Figure 5, resulting in an optimized velocity field that preserves the qualitative structure of the 4D Flow data but at a higher resolution and without visible noise.

Gaidzik et al.^118^ proposed to estimate the amplitude of parabolic velocity boundary conditions in inlet and outlet boundaries for each time instant from 4D Flow data. The inverse method was a variant of the nonlinear ensemble Kalman filter, namely the local ensemble transform Kalman filter (LETKF),^97^ employing a fixed number of 25 particles. Although the Kalman filter assumes that the parameters are time constants—contradicting the pulsating flow rate—the results obtained using a one inlet/one outlet 4D Flow phantom showed good agreement in independent velocity measurements (particle image velocimetry). The framework was later applied to 4D Flow data of a volunteer's Circle of Willies with multiple outlets,^119^ where the accuracy cannot be assessed since no ground truth data was available.

**FIGURE 6:**
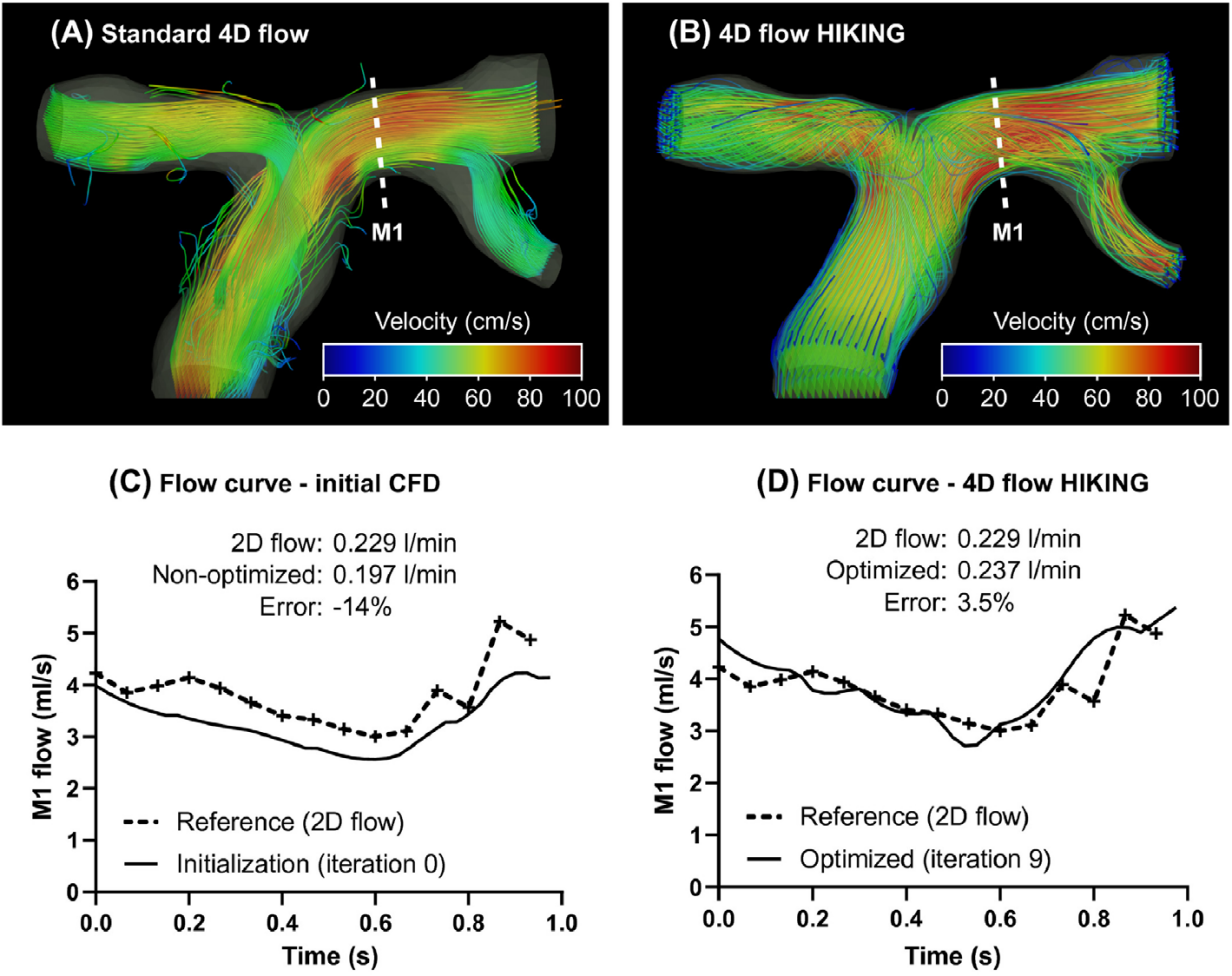
Non‐calibrated flow (A,C) compared to optimized flow simulations (B,D).^120^ (A): streamlines of measured 4D Flow velocities, (B): optimized velocity streamlines, (C): flow rate at cross‐section M1, measured versus computed before optimization, (D): flow rate at cross‐section M1, measured versus optimized solution

Finally, Töger et al.^120^ estimated the Dirichlet velocity data of the pseudo‐compressible Navier–Stokes equations from 4D Flow phantom and patient measurements. In order to reduce the complexity, the boundary velocity profiles were parametrized using few spatial (1 to 3) and few temporal (2 to 12) constants per control boundary, which were simultaneously estimated using a variational data assimilation approach. The authors included also spatial and temporal averaging in the observation operator ℋ as it is present in real 4D Flow data. It was shown that optimizing the boundary conditions using the whole 4D Flow data set—instead of only fitting those parameters from the boundary data—led to an increased precision compared to more accurate measurement modalities like higher resolution 2D PC‐MRI. Those results are shown in Figure 6.

#### 6.4.2 Estimation of boundary data and initial condition

A first, rigorous application of variational DA to the time‐dependent, full Navier–Stokes model was presented by Funke et al.,^121^ adopting a discretize‐then‐optimize approach to simultaneously estimate time‐varying velocity boundary conditions and the uncertain initial condition from velocity measurements. In a synthetic 2D aneurysm, a systematic analysis of the sensitivity of the method with respect to data sparsity, noise, regularization parameters and observation operator (instantaneous vs. time‐averaged) was conducted. The results showed an excellent agreement between reference and DA solution, and proved very robust with respect to the aforementioned factors, given sufficient observations (at least 8 spanning the cardiac cycle) and sufficiently small regularization parameters. As a proof of principle, the methodology was applied to a real 3D aneurysm using 4D Flow ex vivo measurements of a dog's vessel, resulting in more than 200,000 dimensions of the parameter space. In order to make the solution of this challenging problem feasible, relatively coarse numerical resolutions were used to reduce the computational cost. In addition, the viscosity was artificially increased in order to facilitate the numerical solution with the price of introducing a modeling error with respect to the observations. The results agreed qualitatively with the data. This study indicates that variational DA of realistic hemodynamic problems is within reach but emphasizes the present limitations in computational power and efficiency.

**FIGURE 7:**
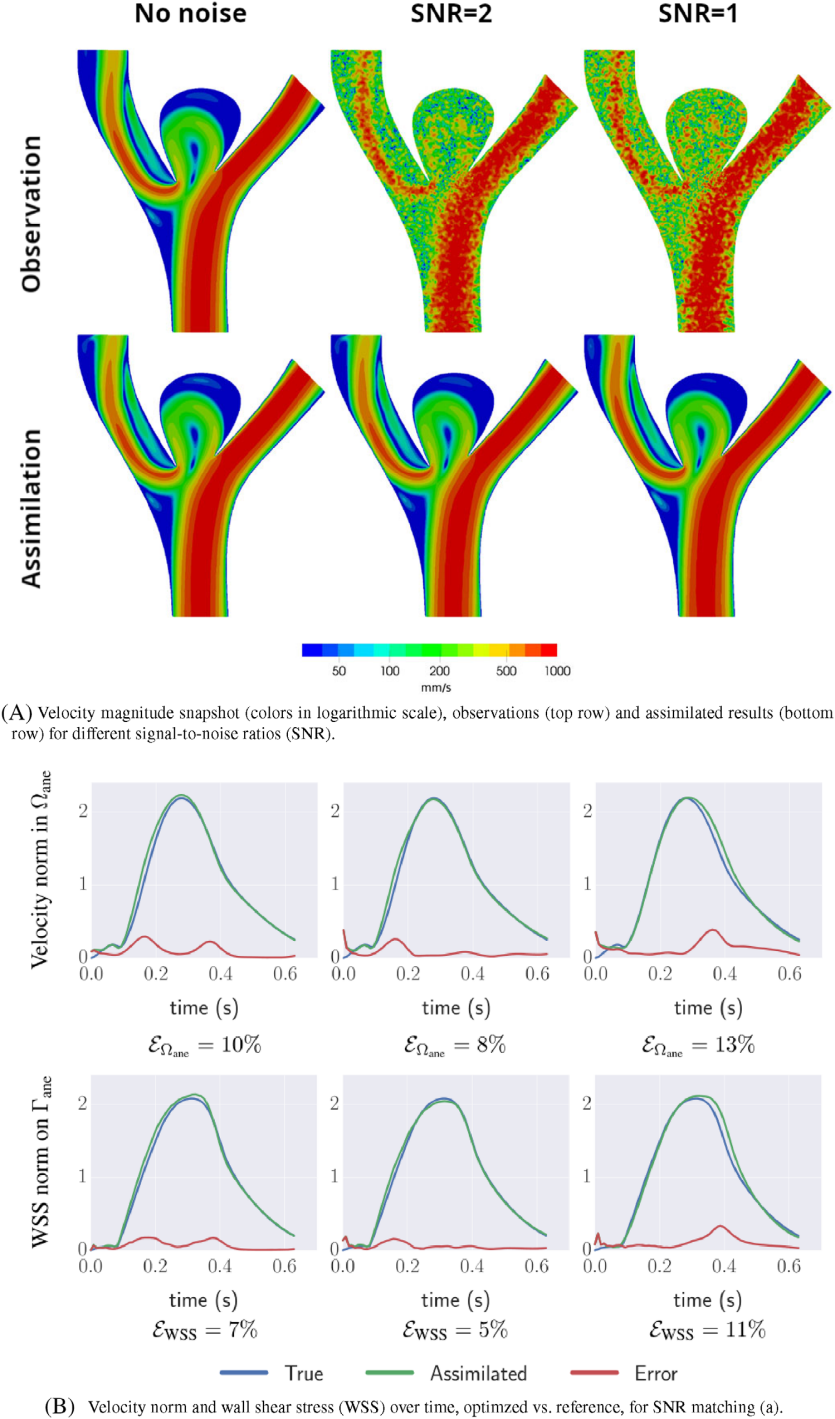
Variational data assimilation results for time‐dependent 2D synthetic data, modified from Funke et al.^121^

2D results of Funke et al.^121^ are displayed in Figure 7, showing the close agreement with the reference solution and the robustness to noise of the data assimilation procedure.

### 6.5 Discussion and perspectives

Approaches based on directly estimating the distributed boundary conditions such as velocity or normal stress data allow for accurate reconstructions of the measured flow dynamics. These approaches can be seen as a way to enrich the data (e.g., high‐resolution reconstructions from sparse data and full‐domain reconstruction when velocity measurements are available in few locations) or to filter/denoise the data using the flow physics (i.e., from noisy 4D Flow measurements).

An open problem seems to be the proof of existence (and uniqueness) of minimizers of the optimization problem in the time‐dependent case. Moreover, the reviewed works have dealt only with rigid wall problems, suggesting that the extension to fluid–structure interaction problems remains an open area of research for both theoretical analysis and computational approaches.

## 7 ESTIMATION OF LUMPED PARAMETER MODELS' CONSTANTS

### 7.1 Preliminaries

The optimized flow models from Section 6 deliver results for a specific flow condition. In contrast, as outlined in Section 2.2, LPMs can yield boundary conditions for flow models which account for the *properties* of the truncated vasculature, represented by model parameters like resistances and compliances. Those parameters can be estimated from data using the methods listed in Section 5, instead of estimating full boundary velocity or normal stress profiles. This considerably reduces the dimension of the optimization problem, allowing also for a reduced amount of measurements. Moreover, this has the advantage that other hemodynamic regimes and physiological conditions can be simulated afterwards, for example, exercise states.

In this section, we structure the review by the type of the methods used to solve the estimation problem, namely variational and sequential data assimilation, and root‐finding approaches.

### 7.2 Variational approaches

Fevola et al.^122^ proposed to estimate a single resistant model (i.e., *C* = 0) on a stationary Stokes problem using variational data assimilation, by means of formulating and solving the continuous adjoint equations. While the inflow profile was directly extracted from the data, measurements of flow rate and average pressure were used in the cost function. Since the resistances of all outlets were optimized simultaneously, the pressure constant of the problem was fixed by pressure discrepancy error in the cost function. It was shown that the flow distribution in the outlets of an ascending aorta obtained with a fully time‐dependent Navier–Stokes model—using the resistances estimated from the stationary Stokes problem—was in close agreement with the one measured in real 4D Flow data sets.

### 7.3 Sequential approaches

In Pant et al.,^33^ the estimation of the LPM parameters in the boundary conditions from flow rate and pressure data was performed using a 0D surrogate models replacing the full multiscale model. The proposed method iterated between the parameter estimation in the 0D surrogate model using a unscented Kalman filter and (few) 3D model evaluations in order to ensure that the 3D model was accurately represented by the surrogate model. Sensitivity analysis of the surrogate model revealed the identifiability of (and combinations of) Windkessel parameters. Tests were shown for real data of carotid arteries and thoracic aorta, also illustrating the capability of data assimilation approaches to predict outputs in different hemodynamic conditions.

**FIGURE 8:**
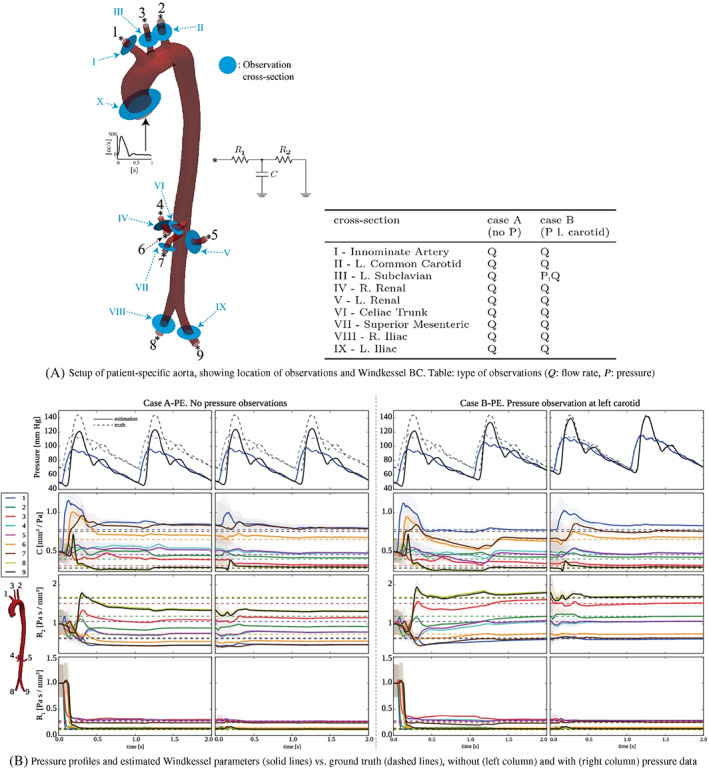
Parameter estimation results of Arthur et al.^123^ using 9 three‐element Windkessel boundary conditions in a patient‐specific aorta from synthetic data. (A) Setting: geometry, observation planes and observed data (cf. table), (B): pressure (at outlets 1 and 9) and WK parameter estimates of cases A and B, with and without pressure observation, compared to the ground truth

Arthurs et al.^123^ applied the Reduced‐order UKF (ROUKF) to the full time‐dependent Navier–Stokes model with simplified fluid–structure interaction (FSI) effects on the wall to estimate the LPM parameters in the boundary conditions. Both time‐varying flow rates—as extracted from 2D PC‐MRI—and pressure forms obtained by applanation tonometry in the carotid were used as measurements in aortas using synthetic and subject's data. In particular, pressure measurements appeared to be crucial to obtain the correct parameters when errors in the pressure's initial condition were present, as is the case in applications with real data. Results of the estimation of 27 Windkessel parameters are shown in Figure 8B, for a patient‐specific aorta with synthetic data given at the observation planes shown in Figure 8A. The results illustrate the beneficial effect of pressure data on the parameter identification and on an accurate recovery of the blood pressure. In the real data case where no ground truth parameters are available, parameters' time evolution during filtering showed a quite stable behavior. Moreover, the model with the optimized parameters matched the flow rate data not used for the estimation well. Both aspects were indicators of probable successful estimation results.

Garay et al.^124^ developed a new cost function for the inverse problem that allows to estimate global fluid parameters when aliasing in the phase‐contrast MRI data is present. The cost function is derived from the original magnetization signal model, hence accounting for the velocity encoding in the phase of the MRI image. Test cases consisted in estimating distal resistances in an aortic geometry from 2D image slices containing the through‐plane velocity component without the need of segmenting the flow rate time curves. This allows to reduce the number of measured slices since the spatial distribution of the velocity improves the identifiably of the parameters. Results showed robust estimation of the resistances up to velocity encoding smaller than 30% of the maximal velocity in synthetic data sets. An example with real data set was shown confirming these results.

### 7.4 Multi‐fidelity global optimization

Perdikaris et al.^125^ proposed to minimize the data discrepancy cost function (i.e. without Tikhonov regularization) to estimate parameters in hemodynamic models using multi‐fidelity, derivative‐free global optimization based on statistical concepts such as Gaussian processes. The application was the estimation of the total resistance (i.e., *R*_*d*_ + *R*_*p*_) from the peak systolic pressure measurement in a 3D Navier–Stokes model using a 1D models as a surrogate. The examples showed that in the case with 2 outlets in the 3D geometry, and hence with 2 parameters to estimate, the parameters can be recovered with about three 3D and eighty‐five 1D model computations. However, it remains unclear how the complexity—and in particular the total number of computations of the 3D model—would scale with the number of parameters to be estimated and/or the complexity of the measurements.

### 7.5 Root‐finding

Spilker and Taylor^126^ estimated Windkessel model parameters for idealized examples and a patient‐specific abdominal aorta, with varied numbers of outlets. Single time independent measurements were considered, such as minimum and maximum inlet pressures, pressure waveform shape indicators and MRI flow rates and flow rate split at bifurcations, representing the target values to be matched by the model in the root‐finding procedure. Their approach, while efficiently delivering accurate results, required accurate initial guesses of the parameters for the quasi‐Newton method (using a finite‐difference approximation of the Jacobian) to succeed. Suitable initial approximations for the parameters and the Jacobian were obtained with the help of a 0D surrogate model in a prior step.

An alternative approach for determining the Jacobian for the Newton method was proposed by Ismail et al.,^127^, ^128^ circumventing the requirement of accurate initial guesses: the Jacobian was obtained by means of the adjoint approach. In contrast to adjoint‐based variational data assimilation, here the Jacobian only considered the LPM, and not the full‐dimensional model, thus vastly reducing the otherwise immense computational cost. Their results showed good convergence, even for inaccurate initial guesses, for different patient‐specific arteries.

Algorithmic improvements to Spilker and Taylor,^126^ addressing the issue of the initial parameters, were introduced by Itu et al.,^129^, ^130^ but only applied in a reduced‐order 1D/0D multiscale approach.

Other iterative approaches have become popular in the literature due to their simplicity. For instance, Troianowski et al.^131^ proposed a simple fixed‐point iteration to estimate the total resistances of 3‐element Windkessel models coupled to the 3D Navier–Stokes equations using measurements of the flow rate splits and of pressure differences between the branches. From the total resistance, the remaining parameters can be estimated according to Spilker et al.^132^ The method was applied to a pulmonary artery, iterating simulations until the computed pressure differences match the measurements with the desired tolerance (errors of ≤1*%* for the pressure gradient and flow were obtained after 3 to 5 iterations). The method was adopted in different studies of pulmonary hemodynamics.^133^, ^134^

Another popular fixed‐point approach is that of Xiao et al.,^135^ who used a surrogate model to iteratively determine the Windkessel parameters of a 3D model coupled with Windkessels at the outflow boundaries. The surrogate model replaced the 3D domain with a 1D model and was coupled to the same 0D Windkessel boundary conditions. In a tailored approach, the Windkessel's total resistance and capacitance were determined by iterations of the surrogate model, until measured pressure values were reached. The converged parameters were then used in the 3D model.

### 7.6 Discussion and perspectives

Root‐finding approaches appear to have been more popular than least‐squares based approaches for estimating LPM parameters in 3D‐0D coupled problems. However, root‐finding approaches have some drawbacks. Most importantly, the number of parameters must match the number of measurements, and therefore it is not obvious how to take into account richer data sources (e.g., time series) and different data types in the root‐finding formulation. In particular in pathological cases, there is no guarantee that the imposed target values will be reached by the model. This is further complicated by noise in the measurements. In addition, root‐finding appears to be very sensitive to the initial guesses and may require special treatment at the startup, as proposed by, for example, Itu et al.^129^

Least‐squares formulations (solved either with variational or sequential approaches) offer better robustness to the aforementioned issues, in particular since they can handle overdetermined problems (i.e., more measurement data points than parameters to estimate), for instance when spatially and/or time‐resolved flow rate/velocity and/or pressure data are available.

Exploiting the reduction of the adjoint to the Windkessel model only, in Ismail et al.^128^ seems to give a reasonably accurate and computationally cheap approximation of the full adjoint. An interesting extension would be to investigate the performance of the reduced adjoint approach in the context of variational data assimilation.

Also, the fixed‐point iterations between a 0D surrogate and the full 3D model in Pant et al.^33^ seems to allow to reduce the number of iterations in the estimation process. An interesting but highly technically complex path to explore in future research to further reduce the number of iterations is by formulating such fixed‐point iterations as root‐finding problems and approximating the Jacobian using composition of operators, as for instance was done in fluid–structure interaction.^136^

## 8 PARAMETER ESTIMATION IN FLUID–STRUCTURE INTERACTION MODELS

### 8.1 Preliminaries

Blood vessels are compliant. The relation between deformation and pressure forces is governed by the parameters of the constitutive model *θ*_*s*_, which have mainly been studied ex vivo, see, for example, Sommer and Holzapfel.^137^ However, it is accepted that the compliance of the artery is an indicator of the subject's cardiovascular health^8^ and therefore there is great interest of in vivo quantification of the constitutive properties.

Since the increase of pressure in systole deforms the vessel, inverse problems can be formulated for estimating the constitutive parameters of fluid–structure interaction (FSI) models from measurements of both the vessel deformation and forces. Due to the complexity of the FSI model, the initial condition is usually not estimated and the models are started “at rest”: zero‐velocity for both fluid and solid. Moreover, the initial displacement is computed by assuming that the geometry Ω is obtained from a segmented image loaded at diastolic pressure, and therefore one should recover zero stress configuration first.^31^, ^138^, ^139^, ^140^, ^141^, ^142^ A simpler alternative is to correct the stresses from the fluid to the solid for all times by the one of the initial simulation time.^43^, ^143^ Note, however, that such an approach allows to estimate only the relative distribution of *incremental* stiffness parameters rather than their absolute values as obtained in ex‐vivo experiments.

Several challenges make the estimation of the arterial wall stiffness difficult. The wall deformation is also constrained by external tissues, for instance the pulmonary trunk in the ascending aorta and the spine in the descending aorta, which however can be represented by elastic Robin boundary conditions with a parameter *α*_*s*_. The heart also imposes a complex motion pattern to the ascending aorta. Last but not least, the arterial wall deformations—in particular in the arch, descending and thoracic aorta—are also small and therefore clinical images like MRI do not provide quality measurements of the wall kinematics. While dynamic CT measurements can be more reliable, they involve larger doses of radiation and are therefore not routinely acquired in the clinical practice.

### 8.2 Arterial wall stiffness

We first review the works presenting estimations of arterial wall stiffness, assuming the external tissue properties are known. The classification is made by the type of DA method used.

#### 8.2.1 Variational approaches

Perego et al.^144^ introduced a variational approach, using a DO strategy, in order to estimate the Young's modulus of a linear elastic solid from displacement measurements at the fluid–solid interface. The problem was formulated as stationary minimization approach at each time step, with the Young's modulus as the only control parameter. The existence of minimizers of the problem was proved. In the numerical algorithm, the estimated Young's modulus for each time step served as initial guess for the estimation in the next time step, hence avoiding the solution of challenging time‐adjoint problems. The final estimate consisted in an average of the estimates over time. Numerical tests with synthetic noisy data in 2D showed good agreements of the ground truth with the estimated parameters in spite of the measurement noise. However, the authors suggested that solving for each time step independently—not considering the information of the previous solution—may suffer instabilities in the presence of highly noisy data.

Bertagna and Veneziani^145^ further investigated how to accelerate the computations in the case that the solid is treated as a thin membrane. The approach was based by pre‐computing a series of solutions for different Young's modulus and building a proper orthogonal decomposition (POD) basis, hence considerably accelerating the linear system solutions during the optimization. Synthetic 3D examples with three Young's modulus were presented in two geometries and different noise levels. For those cases, however, in turned out that the total number of linear solves was only slightly reduced compared with the case without POD. The authors discussed at the end different strategies on how to further accelerate the computations.

#### 8.2.2 Sequential approaches

The first results on sequential estimation of parameters in FSI problems were reported by Bertoglio et al.^143^ The methodology used was a ROUKF for the estimation of the distribution of the Young's modulus from Lagrangian wall displacement measurements. The accuracy of the estimated parameters depended on the displacement‐to‐noise ratio. Additionally, it was shown that it is possible to identify the Windkessel resistance from the same measurements, when the wall stiffness is known. These results were extended in the case of an elastic tube in Arthurs et al.,^123^ where the arterial wall stiffness together with Windkessel parameters were estimated from flow rate, pressure and wall displacement data.

**FIGURE 9:**
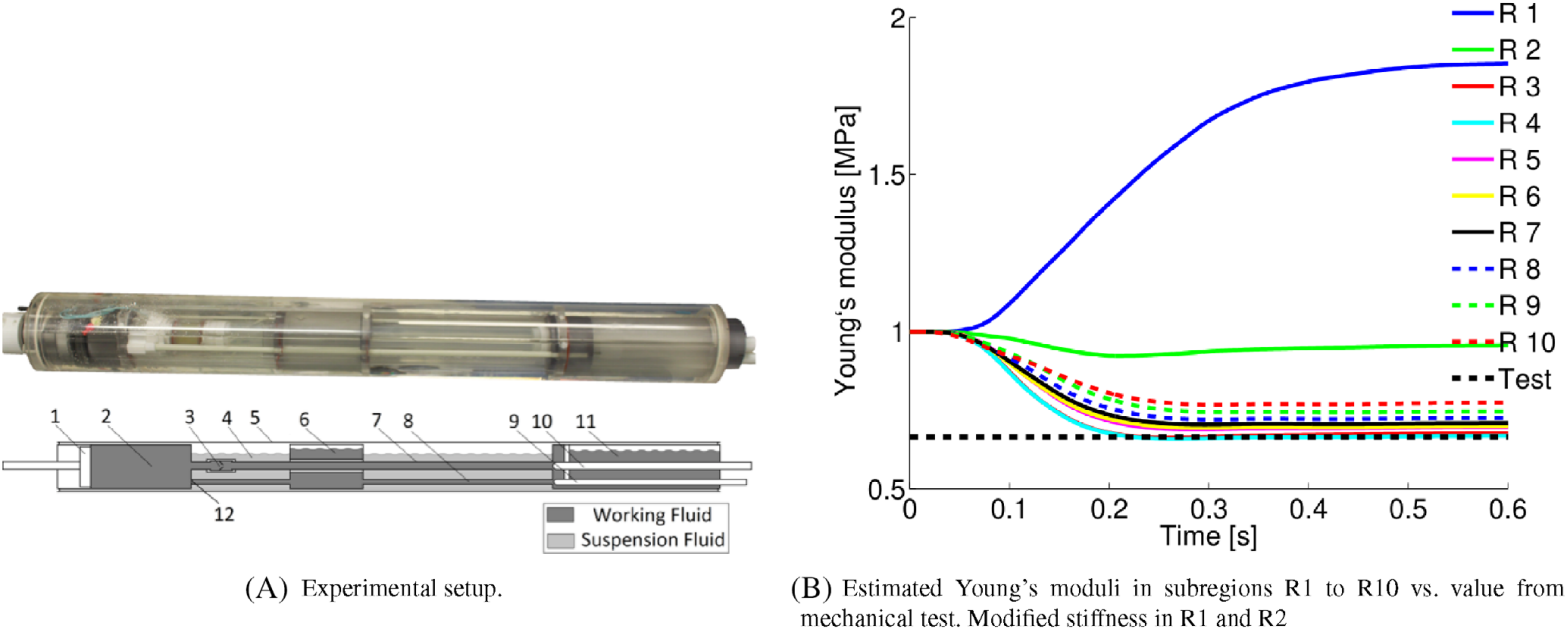
FSI wall stiffness (Young's modulus) estimation setup and results from Bertoglio et al.^43^ Reprinted with permission

The ROUKF approach was validated with both phantom and volunteer MRI data in Bertoglio et al.^43^ In the experimental validation, a silicon rubber tube was considered, connected with mechanical emulator of the cardiovascular system (Figure 9A). An additional elastic patch was included in one of the segments for inducing a local stiffening. The FSI computational model of the cylinder was set up based on the data segmented at one time instant. Pressure measurements were used for the fluid boundary conditions. The Young's modulus along ten sub‐segments of the tube was estimated from surface measurements from segmentations of dynamic multi‐slice MR images. The estimated Young's modulus for the all segments matched the value obtained from a mechanical test, cf. Figure 9B. The location of the additional elastic patch showed an increased stiffness (*R*1, *R*2 in Figure 9B).

Also in Bertoglio et al.,^43^ the ROUKF was then assessed for estimating the Mooney–Rivlin constitutive parameters distribution (five sub‐regions) in a patient with repaired aortic coarctation. The measurements used were segmented dynamic anatomical MRI images and therefore the observation operator consisted in distance maps from the model to the segmented surfaces. While the model with estimated parameters followed closer the segmented surfaces, the stiffness was estimated larger in the regions where the aorta was in contact with surrounding vessels due to the absence of modeling of that contact.

#### 8.2.3 Some additional remarks

There exist other approaches that allow to obtain stiffness estimations of vessels in vivo. When invasive pressure catheterization and images of the deformation of the cross sections are available, localized stiffness measurements are possible, see, for example, Stalhand.^146^ A more advanced approach consists in computing the vessel stiffness from deformation maps obtained from dynamic CT and from non‐invasive sphygmomanometer pressure measurements.^11^ The latter, however, assumes a linear material and neglects 3D deformation effects.

### 8.3 Boundary tissue support

The inclusion of the external tissue effect on the aorta was introduced in Moireau et al.^31^ through the application of viscoelastic Robin boundary conditions in FSI models. The estimation of the elastic Robin parameter *α*_*s*_ from wall displacement data—assuming the arterial wall stiffness is known—has been performed using first sequential and later variational approaches.

#### 8.3.1 Sequential approaches

**FIGURE 10:**
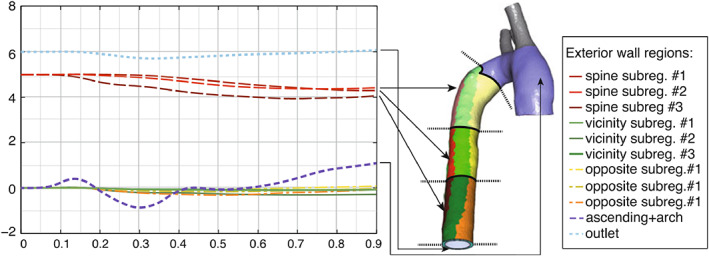
External tissue parameter identification for a patient‐specific aorta with real data. Initial guesses from prior estimation with four tissue regions. Reprinted with permission from Moireau et al.^147^

In Moireau et al.^147^ the parameter *α*_*s*_ in an FSI model of an aorta was estimated using ROUKF from segmented dynamic CT images. The external surface of the aortic model was divided mainly into ascending aorta, arch, supra‐aortic branches, while the descending aorta was subdivided into spine, spine vicinity and opposite to spine. On each surface region a different Robin stiffness value to be estimated was assigned. Distance maps of model‐to‐image contours were considered to quantify the discrepancy between modeled and segmented surfaces. Here, the motion of the aortic root is of great importance and was also included in the model of the ascending aorta using a Lagrangian tracking approach. The results showed that the model including the optimized coefficients reproduced the measured arterial wall dynamics from the CT better than without taking into account the external tissue effect. Results obtained in a patient‐specific setting with real data are shown in Figure 10, where a prior estimation with 4 tissue regions was further refined to 11 regions with distinct parameters, all showing stable convergence except the parameter representing the aortic arch.

#### 8.3.2 Variational approaches

Pozzi et al.^148^ estimated the elastic tissue support parameter *α*_*s*_ for the carotid artery to emulate the effect of arteriosclerotic plaque. In order to characterize the plaque and other surrounding tissues, such as the nearby jugular vein, the elastic parameters of the boundary condition were spatially distributed and their values were estimated by minimizing the discrepancies between computed vessel displacements and reference values obtained from segmented surfaces from dynamic MRI data. The optimization procedure was performed using a derivate‐free approach, hence no adjoints were implemented. The results showed that the surrogate model via the optimized Robin boundary conditions is closer to a full FSI model of the carotid plaque than a rigid walls simulation.

### 8.4 Discussion and perspectives

One important open problem is the joint estimation of arterial wall stiffness and boundary support parameters. It is accepted that the bottleneck comes from the fact that standard measurements (like pure surface distances) are not enough to distinguish the effects of both parameters.

Another bottleneck is the quality of data. While MRI measurements of the arterial wall motion appeared to be poor due to imaging and flow artifacts and sometimes subvoxel motion of the wall, dynamic CT images are not used in clinics since they require about the annual recommended total radiation dose.^39^ Including additional measurements in the stiffness estimation (e.g., flow velocities in MRI) could compensate for the poor data quality of the arterial wall displacements.

It is also important to mention that usually only one of the constitutive parameters is estimated in vivo, in contrast to the ex vivo setup. Estimating all arterial wall constitutive parameters requires measuring forces and deformations in all directions. However, in vivo only the surfaces and not the full arterial wall displacement field ***d***_*s*_ can be tracked, and loading is dominated by pressure (normal) forces.

## 9 COMPENSATING ERRORS IN THE COMPUTATIONAL DOMAIN

### 9.1 Preliminaries

Most, if not all, personalized models assume that the computational domain Ω is not afflicted by measurement errors and uncertainty, but make the assumption, that the vessel wall is segmented at the correct position. However, the segmentation process used to determine the vessel contours is subject to errors caused by the limited image resolution, flow artifacts and partial volume effects.^149^ A detailed analysis of the impact of geometric uncertainties in a stenosed anatomy on the pressure difference was carried out by Brüning et al.^66^ and on wall shear stress by Perinajová.^150^ Recently, the issue of geometric errors was investigated theoretically and error bounds were presented.^151^ All studies found that small changes in geometry cause important variations in the solution.

### 9.2 Compensation using velocity measurements

Additional velocity measurements have been used to estimate boundary conditions on the part of the boundary which the vessel wall should be present, hence moving out from the homogeneous Dirichlet data assumption.

**FIGURE 11:**
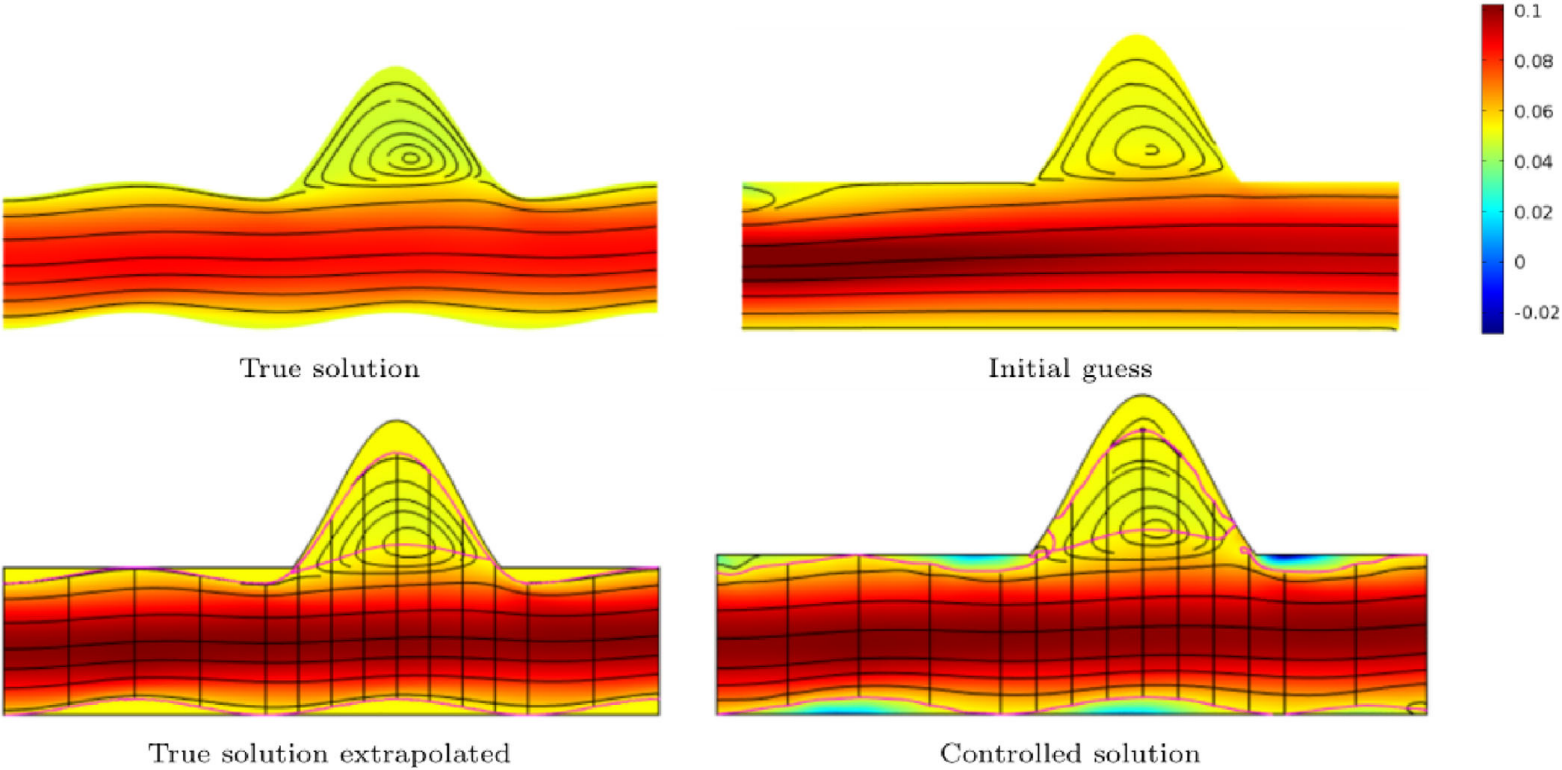
Geometry error compensation by means of boundary velocity optimization.^111^ Plots show velocity streamlines and velocity magnitude. The zero‐velocity streamline is plotted in magenta in the bottom row plots, approximating the true domain. Reprinted with permission

The data assimilation approach of Tiago et al.,^111^ reviewed in Section 6.3, also addressed the case where the computational and the real domain do not match by estimating the non‐zero Dirichlet boundary conditions on the boundary, thus approximating the vessel wall position. 2D tests with synthetic measurements showed good agreements between the reference (computed in the true domain) and the estimated solution—see Figure 11. This approach requires however full field velocity measurements (like 4D Flow).

A simpler approach was introduced by Nolte and Bertoglio^152^ where it was proposed to augment the flow model calibration by slip/transpiration boundary conditions, whose parameters are then estimated using velocity measurements. This approach is exact in a developed pipe flow. For a more general flow, the slip and transpiration parameters can be assumed as space–time constants, which leads to a more tractable inverse problem than estimating the spatial velocity profile as in Tiago et al.^111^ The additional advantage of the reduced dimensionality is that a smaller amount of measurements, for example, 2D slices with velocity information in one direction only, suffices to estimate those parameters and hence to compensate for the geometrical errors. Numerical tests using 3D synthetic Navier–Stokes flows showed that including an additional 2D slice allows for important increases in robustness of computed quantities like pressure drops across stenotic vessels. While the solution method was the ROUKF, a variational approach could have also been used.

**FIGURE 12:**
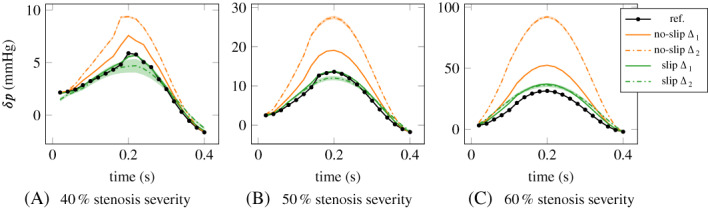
Pressure differences across 3D synthetic stenoses over time, with optimized slip/transpiration (˝slip˝) versus no‐slip boundary conditions for different stenosis severities. Mean values with ±2*σ* bands over 30 samples of partial noisy measurements, given at the inlet and in one interior plane, with data resolution/geometry error Δ_1_ = 1 mm and Δ_2_ = 2 mm. Reprinted from Nolte and Bertoglio^152^

Figure 12 illustrates for an idealized 3D stenosis with synthetic data the accuracy of the pressure differences across the stenosis, obtained on a domain with erroneous wall locations using no‐slip and optimized slip/transpiration conditions. The optimization estimated the unknown amplitude of a pulsatile inflow and two slip/transpiration parameters, constant in time and space, from synthetic 2D MRI flow data. The agreement between the ground‐truth reference solution and slip//transpiration model is significantly more accurate than standard no‐slip boundary conditions.

### 9.3 Discussion and perspectives

It is still an open problem to extend these approaches to—or develop new ones for—the FSI case. While if full field velocity measurements are available the approach of Tiago et al.^111^ allows to solve for the fluid only—implicitly considering the arterial wall kinematics from the data—in the case of FSI it is an open problem how the coupling conditions should be formulated.

## 10 STATE ESTIMATION

### 10.1 Preliminaries

Blood flows are time‐dependent due to the pulsatile nature of the cardiovascular physiology. Therefore, dealing with time‐dependent effects is important. The evolution of the flow dynamics essentially depends on the initial condition, *X*(*t*_0_), which in the general case is unknown and has to be estimated from data. The present section reviews the literature addressing the issue of state estimation under uncertain initial conditions, again structured by the optimization method employed.

### 10.2 Rigid‐wall models

#### 10.2.1 Variational approaches

To the best of our knowledge, the only work fully estimating the initial condition in blood flows is Funke et al.,^121^ using variational DA and simultaneously estimating the time‐varying velocity boundary conditions. A detailed discussion of their work was already presented in the context of boundary condition estimation, cf. Section 6.4.2.

#### 10.2.2 Kalman filter approaches

In Habibi et al.,^153^ a linear Kalman filter was applied to estimate the state from synthetic 4D Flow data in aneurysm geometry. For the filter to become tractable, the system dynamics were reduced using dynamic mode decomposition^154^ generated with errors of about 10% in the inflow velocity and viscosity. In 3D, improvements with respect to the ground truth were about 70% compared to the non‐filtered case.

#### 10.2.3 Luenberger observer approaches

Velocity feedbacks were proposed in Funamoto et al.^155^, ^156^ in the context of blood flows, using Doppler‐like synthetic velocity measurements. The study, however, did not assess recovering the state trajectory from an uncertain initial condition but by setting up the observer with incorrect boundary conditions. It was shown that the observer could also reduce the error with respect to the reference simulation in that situation.

An important remark is that velocity observers in incompressible flows based on localized velocity measurements (e.g., slices) is that the effect of the feedback is localized around the measurement location due to the parabolic properties of the fluid system.^157^ Therefore, effective fluid observers based on velocity measurements should be ideally based on full field measurements such as 4D Flow.

Additionally, observers in incompressible flow can only be proven to reduce the velocity error in the energy norm, hence there is no control on the pressure error. Indeed, Funamoto and Hayase^158^ showed numerically that velocity observers may produce spurious pressures. Also, a pressure compensation method was devised where the observer's pressure was corrected a posteriori in the regions where the divergence of the feedback term is large.^158^ In order words, this is equivalent to taking only the divergence‐free part of the feedback term in order to avoid pressure perturbations. Results showed to be more accurate than the approach without any correction from Funamoto et al.^156^ In Ii et al.,^159^ the approach was further refined to applying then divergence‐free feedback term only at inlet and outlet boundaries, showing that the observer presents reduced perturbations from to the measurement noise with respect to the feedback applied on the whole domain. As the authors claimed, theoretical analysis that supports the theoretical results remains an open task.

### 10.3 Fluid–structure interaction models

There is only one reported work on state estimation in FSI problems,^160^ where the performance of the observers presented in Moireau et al.^161^, ^162^ based on displacement and velocity measurements in the solid was studied theoretically and numerically in the FSI framework. It was shown that the straightforward usage of the these estimators in FSI leads to a considerably better performance of the displacement with respect to the velocity feedback, while in pure solid mechanics usually the opposite occurs. The theoretical analysis concluded that the velocity feedback does not take into account (by construction) the added mass effect in the FSI case. Hence, an improved observer was proposed by including the added mass operator in the feedback. It was also shown that to accelerate the convergence of the observer to the target trajectory for the full fluid–solid state also velocity measurements in the fluid are needed.

### 10.4 Discussion and perspectives

Independent of the method, ideally both initial condition and parameters should be estimated. In the context of variational data assimilation, incorporating the initial condition seems still to be an open problem for FSI problems. The methods and theory developed by Failer^163^ for the control of FSI problems can be extended to tackle the initial condition estimation.

As mentioned in Section 5.3, the full estimation of the initial condition with a Kalman filter is computationally prohibitive. An approach to partially tackle this issue is to assume that the initial condition belongs to some (linear) subspace of reduced dimension, and include those degrees of freedom as uncertain parameters.^164^

Joint parameter and state estimation can also be performed by combining Luenberger observers and Kalman filters, as was successfully done in solid mechanics.^104^ In Luenberger observers with perfectly known parameters, the error in the whole state in the energy norm decreases over time. However, in multi‐physics problems when (partial) observations in only one of the fields are available, it may happen that the error in the fields which are not measured increases. This may negatively impact the estimation of the parameters when Luenberger and Kalman filters are combined.

Another challenge to be addressed in Luenberger observer problems in FSI is the choice of the scalar gain within ℒ, since it results from computationally expensive eigenvalue.

## 11 FURTHER TOPICS

The literature reviewed in this article was limited to settings considering the full 3D/2D models and the estimation of their material properties, boundary conditions and initial conditions. In this section, we list some of the works dealing with related topics, without presenting exhaustive commentary. We believe that this may be of interest to the readers of this article.

### 11.1 Inverse uncertainty quantification

Bayesian parameter estimation—as given in Section 5.1—enables uncertainty quantification (UQ) of the parameter estimates, as parameters and the corresponding model outputs are considered probabilistic distributions.

It should be first noted that parameter estimation based on Kalman filters (references^31^, ^33^, ^43^, ^104^, ^118^, ^119^, ^123^, ^124^, ^143^, ^146^, ^151^, ^152^ in Sections 6 to 9, and references^34^, ^164^, ^165^, ^166^, ^167^, ^168^, ^169^ for GROMs in Section 11.4) inherently includes uncertainty quantification via tracking of the covariance matrix (following an assumption of Gaussian distributions) of the parameters. As follows, we will review research based on other types of solution methods for the Bayesian estimation problem.

Lassila et al.^171^ presented methods of computational and geometric order reduction which were applied to deterministic and Bayesian parameter estimation problems. For a steady FSI model of a 2D idealized stenotic vessel, employing a reduced basis, parameter distributions of the elastic wall model were inferred from measurements of the pressure drop. The Young's modulus could be reliably identified. The estimation proved very sensitive to noise. Despite the shear modulus resulting to be unidentifiable, distributions of the wall shear stress could be estimated from pressure drop data by means of Monte Carlo sampling. A second example explores optimal bypass design for a occluded femoral artery and estimates the residual flow distribution from pressure drop measurements.

Further publications study inverse UQ in geometrically reduced problems (see also Section 11.4), and shall be listed only briefly and with no claim to completeness. Considering 1D arterial networks, Arnold et al.^172^ studied the estimation of inflow waveforms from ex vivo pressure and vessel area measurements in a probabilistic setting using the EnKF and providing UQ for the inflow, pressure and vessel area estimates. Paun et al.^173^, ^174^ investigated Bayesian parameter estimation and UQ using Markov Chain Monte Carlo sampling and Gaussian processes for 1D models of the pulmonary arteries in mice. Arterial wall stiffness and Windkessel boundary parameters were inferred from in vivo and synthetic data. Schiavazzi et al.^175^ perform UQ in the context of virtual heart surgery. First using an LPM, Bayesian inversion relates uncertain clinical data on the pressure and flow split ratio with Windkessel parameter output distributions. In the second step consists in the forward propagation of the parameter uncertainty with a 3D Navier–Stokes model, where the Windkessel parameters enter as boundary conditions. Similarly, in Tran et al.,^176^ Bayesian parameter estimation problems were solved via Monte Carlo sampling in LPMs of coronary arteries, to recover hemodynamic parameter distributions from non‐invasive clinical data. These were in a separate step propagated with a full multiscale model to obtain confidence intervals of 3D results. Larson et al.^177^ formulated a Bayesian parameter estimation and UQ framework for bifurcating arterial 1D networks. Given noisy, synthetic velocity observations, they estimated the blood viscosity, arterial stiffness, and vessel area, and identify structural defects (modeled via perturbations in the latter two parameters).

### 11.2 State estimation by linear optimization

Rispoli et al.^178^ presented a linear least squares method to denoise and increase the spatial resolution of 4D Flow data. The velocity estimate is the one minimizing the weighted sum of two quadratic terms: (a) the discrepancy between the velocity estimate and 4D Flow data and (b) a term that penalizes the Navier–Stokes model residual using a time‐explicit discretization. The velocity estimate results in a weighting of a Navier–Stokes solution and the measured data. The approach may therefore be interpreted as data denoising or model correction, depending on the values of the weights.

With a similar purpose a different approach was presented by Fathi et al.^179^ A forward Navier–Stokes simulation with boundary conditions based on the 4D Flow rates served to generate snapshots from which a reduced basis was constructed using *proper orthogonal decomposition* (POD). A velocity estimate in the POD basis was found by balancing the quadratic error with respect to 4D Flow measurements and a lasso *ℓ*_1_ regularization promoting sparsity in the POD basis. Comparisons for in vivo 4D Flow data to other denoising methods showed a superior performance of the POD‐lasso approach. However, the question of the choice of the penalization parameter of the *ℓ*_1_ term remains open.

With the aim of extrapolating 2D measurements to the whole 3D domain using the flow physics, Galarce et al.^180^, ^181^ proposed an approach to find the element of a linear subspace (e.g., POD basis) closest to the true 3D flow velocity solution such that the estimate matches the measurements. This match can be performed exactly or in a least squares sense. The number of elements of the POD basis was chosen such that a priori error bounds for a quantity of interest of the flow (velocity, pressure drop) were minimized. Moreover, an inequality constraint on the basis coefficients was included, avoiding penalization parameters in the optimization functional. The approach was extended in Galarce et al.^182^ to account for domain shape uncertainties when the domain's geometry is not easily parametrizable.

It is interesting to remark that the formulations presented in these works^179^, ^180^, ^181^ are complementary, for instance by combining the *ℓ*_1_ regularization^179^ with the a priori mode selection and coefficient constraints.^180^, ^181^ Concerning the computational cost, both cases are dominated by the number of forward solutions. In Fathi et al.,^179^ only one forward simulation was used to generate the snapshots for the POD basis because the boundary conditions were extracted from the 4D Flow data. Since in Galarce et al.^180^, ^181^ only a smaller number of (sparse) measurements was assumed (like 2D Doppler ultrasonography images), the basis was generated by probing the flow's model parameter space (e.g., boundary conditions) leading to an important number of forward computations. However, the strategy for generating snapshots is independent on the estimation method and rather depends on the data availability.

All the works mentioned in this section have focused on rigid wall models, and therefore their extension to FSI remains to be investigated.

### 11.3 Machine learning

Machine learning techniques, in particular ˝deep learning˝ algorithms using deep neural networks (DNN), have made their entry into the field of computational medicine during the last decade. Publications on deep learning in medical image analysis seem to be growing exponentially and numerous recent and heavily cited literature surveys^183^, ^184^, ^185^, ^186^, ^187^ have appeared, trying to keep track of the dynamic developments. Deep learning is already being employed for the classification of exams and image features, for the detection and location of organs and lesions, has become the gold standard in image segmentation, is used for registration and other purposes.^183^ Related to image‐based hemodynamic analysis, first proofs of concept propose deep learning‐based data‐driven surrogate models for aortic flows,^188^, ^189^, ^190^ which have the potential to become useful in the context of inverse hemodynamics in the future. However, research of machine learning techniques to solve inverse problems in hemodynamics remains scarce, although a growing number of publication can be expected following the general trend in other fields.

An approach to include physical models in neural networks was proposed by Raissi et al.,^191^ deviating from the purely data‐driven paradigm of machine learning. Their physics‐informed neural networks (PINN) enable state estimation and parameter identification. Making use of the universal function approximator property of DNNs, functions (e.g., blood velocity) are expressed as DNNs that take time and space coordinates as inputs. Automatic differentiation of DNNs allows evaluating the residual of PDEs governing the approximated functions, which is minimized during the learning process of the DNNs. Raissi et al.^192^ used a PINN to accurately recover the velocity and pressure fields in a 3D intracranial aneurysm from observations of a scalar tracer concentration, using synthetic data. An advantage over patient‐specific simulations is that no flow boundary conditions are required. Kissas et al.^193^ employed PINNs to predict the velocity and pressure wave propagation in a 1D compliant vessel model from 4D Flow velocity and wall displacement measurements. Their method avoids mesh generation and the prescription of boundary conditions. It requires large per‐patient training sets, which could possibly be alleviated by transfer learning methods. Fathi et al.^194^ explored the reconstruction of a denoised, high‐resolution flow velocity from 4D Flow data using a physics‐informed DNN following Raissi et al.,^191^ adapted to the specific properties of 4D Flow data acquisition. An in vitro validation using high‐resolution PIV measurements as reference showed a qualitative improvement compared to the input 4D Flow data, but no significant improvement in the error metrics.

In addition, the PINN framework allows parameter estimation,^191^ but this feature has not yet been applied to problems in hemodynamics. Regazzoni et al.^195^ combine data assimilation of cardiovascular flow (a two‐stage Windkessel model) with machine learning to identify an evolution law of unobservable, slowly evolving parameters (Windkessel resistances). A neural network is used to express the right hand side of the evolution ODE and calibrated by means of adjoint‐based solution of a minimization problem. The framework is applied to a data‐driven model of hypertension development in a synthetic, idealized setting and provides accurate results.

### 11.4 Calibration of stand‐alone lumped‐parameter and 1D models

When flow rates are available at each of the outlets of a 3D geometry, and some information is given on the pressure wave—i.e., either from invasive measurements or an assumed wave form calibrated using non‐invasive diastolic/systolic cuff measurements—the LPM parameters can be estimated independently for each of the outlet, as, for example, done by Romarowski et al.^196^

In cases where data such as flow rates is distributed over the arterial tree, data assimilation techniques for the calibration of 1D networks or LPMs of (parts of) the cardiovascular system have been applied. Here, we briefly summarize the literature, with no claim to completeness.

Single or coupled multicompartment LPMs—governed by ODEs independent of spatial coordinates—have been calibrated with measured flow rates and pressure data traditionally by means of nonlinear least‐squares optimization, following the early works^197^, ^198^, ^199^ of the 1980s. Since then, similar approaches, successively refined, have been used in LPMs of the cardiovascular system.^200^, ^201^, ^202^, ^203^, ^204^

Schiavazzi et al.^175^, ^205^ and Tran et al.^176^ used Bayesian estimation approaches (Markov Chain Monte Carlo) for the identification of parameters in complex circulation models, while Pant et al.^165^, ^166^ performed sequential data assimilation in form of the UKF.

Parameter estimation in 1D GROMs, modeling the cardiovascular system as a network of compliant pipes, was undertaken by, for example, Cousins and Gremaud^206^ using least‐squares optimization and special structured‐tree boundary conditions^207^ and by Blanco et al.^208^ by means of a root‐finding problem. Ventre et al.^209^ and Carson et al.^210^ calibrate staged 0D‐1D approaches via least‐squares optimization and root‐finding, respectively. More flexibility regarding parameters and data is offered by sequential data assimilation, for example, by means of the EnKF^167^, ^168^ or the ROUKF.^34^, ^169^, ^170^ Bayesian parameter estimation was conducted in several publications,^172^, ^173^, ^174^, ^177^ briefly discussed in Section 11.1.

### 11.5 Shape estimation of vessels and obstacles

As indicated in Section 9, velocity measurements allow to estimate the shape of obstacles within a vessel. This can be an interesting approach for instance for cardiac valve or stents, which are hard to visualize in non‐ionizing images like MRI.

This problem can be tackled by shape optimization approaches (see, e.g., Quarteroni and Rozza,^211^ Agoshkov et al.^212^ and Manzoni and Ponti^213^). It is important to notice that shape optimization, when the domain is parametrizable, can be accelerated using model reduction techniques, as done in Lassila et al.^171^ for the case of design of bypass grafts.

A simpler approach was presented in Aguayo et al.^214^ and applied to cardiac valves by means of estimating a resistance function approximating the obstacle in the stationary Navier–Stokes equations from velocity measurements.

## Data Availability

Data sharing is not applicable to this article as no new data were created or analyzed in this study.
